# Murine tribbles homolog 2 deficiency affects erythroid progenitor development and confers macrocytic anemia on mice

**DOI:** 10.1038/srep31444

**Published:** 2016-08-23

**Authors:** Kou-Ray Lin, Hsin-Fang Yang-Yen, Huang-Wei Lien, Wei-Hao Liao, Chang-Jen Huang, Liang-In Lin, Chung-Leung Li, Jeffrey Jong-Young Yen

**Affiliations:** 1 Institute of Biomedical Sciences, Academia Sinica, Taipei, Taiwan; 2 Taiwan Mouse Clinic, Academia Sinica, Taipei, Taiwan; 3 Institute of Molecular Biology, Academia Sinica, Taipei, Taiwan; 4 Institute of Cellular and Organismic Biology, Academia Sinica, Taipei, Taiwan; 5 Institute of Biological Chemistry, Academia Sinica, Taipei, Taiwan; 6 Department of Clinical Laboratory Science and Medical Biotechnology, School of Medicine, National Taiwan University, Taipei, Taiwan; 7 Genomic Research Center, Academia Sinica, Taipei, Taiwan; 8 Institute of Molecular Medicine, School of Medicine, National Taiwan University, Taipei, Taiwan

## Abstract

Tribbles homolog 2 (Trib2) is a member of Tribbles protein pseudokinases and involves in apoptosis, autoimmunity, cancer, leukemia and erythropoiesis, however, the physiological function of Trib2 in hematopoietic system remains to be elucidated. Here, we report that *Trib2* knockout (KO) mice manifest macrocytic anemia and increase of T lymphocytes. Although Trib2 deficient RBCs have similar half-life as the control RBCs, *Trib2* KO mice are highly vulnerable to oxidant-induced hemolysis. Endogenous *Trib2* mRNA is expressed in early hematopoietic progenitors, erythroid precursors, and lymphoid lineages, but not in mature RBCs, myeloid progenitors and granulocytes. Consistently, flow cytometric analysis and *in vitro* colony forming assay revealed that deletion of *Trib2* mainly affected erythroid lineage development, and had no effect on either granulocyte or megakaryocyte lineages in bone marrow. Furthermore, a genetic approach using double knockout of *Trib2* and *C/ebp*α genes in mice suggested that Trib2 promotes erythropoiesis independent of C/ebpα proteins *in vivo*. Finally, ectopic expression of human Trib2 in zebrafish embryos resulted in increased expression of erythropoiesis-related genes and of hemoglobin. Taking all data together, our results suggest that Trib2 positively promotes early erythrocyte differentiation and is essential for tolerance to hemolysis.

Depending on the timely expression of regulatory transcription factor genes, hematopoietic stem cells in bone marrow (BM) have the capability of either long-term self-renewal or differentiation into various cell types within the hematopoietic population. Numerous transcription factors are specifically expressed in certain cell lineages and are responsible for their differentiation and maturation. During myeloid cell development, the transcription factor C/ebpα is highly expressed in early myeloid progenitors and mature granulocytic cells and regulates expression of numerous myeloid genes^1^. Knockout of *C/ebp*α blocks granulocyte differentiation in fetal liver and newborns, suggesting that C/ebpα is a key factor for granulopoiesis^2^. Ectopic expression of C/ebpα induces granulocyte differentiation of hematopoietic progenitor cell lines^3^ and promotes neutrophil maturation at the expense of macrophage development in *pu.1*^−/−^ cells^4^, indicating that C/ebpα also plays a role in lineage specification of committed granulocyte-macrophage progenitors (GMPs). Furthermore, deletion of *C/ebp*α increases the numbers of erythroid progenitors and erythroid cells in the *C/ebp*α^−/−^ mouse fetal liver, and enforced expression of C/ebpα in hematopoietic stem cells (HSCs) reduces the number of erythroid progenitors and induces myeloid development^5^. In accordance with these observations, expression of erythroid-specific genes is inhibited by enforced expression of C/ebpα in murine erythroleukemia cells^5^. Additionally, expression of megakaryocyte lineage–associated genes and regulators of megakaryocyte/erythroid (Mk/E) lineage potential, including Gata-1 and Fog-1, are upregulated in the BM of *C/ebp*α^−/−^ adult mice^6^. Together, these data strongly suggest that C/ebpα plays an important role in determining lineage selection between myeloid and Mk/E cell lineages in more primitive progenitors.

Conversely, the transcription factor Gata-1 has long been recognized as a key factor in erythropoiesis, and *Gata-1*-deficient embryos die at embryonic day 10.5 owing to defective erythropoiesis^7^. Conditional deletion of *Gata-1* in adult mice results in depletion of the erythroid compartment in BM and spleen^8^, and the numbers of burst-forming units-erythroid (BFU-E) and colony-forming units-erythroid (CFU-E) in BM are significantly reduced^5^. Recently, with the identification and isolation of bipotential Mk/E progenitors (pre-MegEs), unipotential megakaryocyte and erythrocyte progenitors, and an early myeloid committed progenitor^9^, the erythroid transcription factor Gata-1 has been recognized as essential for erythroid lineage commitment, whereas Fog-1 — a transcriptional co-activator of Gata-1 — is required for development of all megakaryocyte- and erythrocyte-lineage progenitors^6^. A *Fog-1*-deficient bipotential MEP has been reported to undergo myeloid differentiation but not erythroid and megakaryocytic differentiation, as exhibited by wild-type control cells^6^. Of note, both C/ebps and Fog-1 show transcriptional cross-regulation during a period of early myelo-erythroid lineage determination, suggesting this transcriptional antagonism may be the mechanism that accounts for separation of the myeloid and Mk/E lineages^6^. While searching for the putative regulator of Fog-1-dependent extinction of myeloid potential in MEP cells, *tribbles homolog 2* (*Trib2*) was shown to be highly expressed in wild-type MEPs but significantly reduced in *Fog-1*^−/−^ MEPs, suggesting its involvement in Mk/E lineage determination^6^.

Trib2 is a tribbles family member of protein serine/threonine pseudokinases, and its expression in hematopoietic and lymphoid cells is inducible by cytokine starvation^10^. Recently, the tribbles pseudokinase is no longer regarded as a dead enzyme and evolutionary relics as three mammalian tribbles family members, Trib1, Trib2 and Trib3, are all reported to play important role in signaling transduction, via their interaction with various kinases, ubiquitin ligases, and key transcription factors. Many human diseases are also implicated with the overexpression of tribbles family members^11^. Especially, Trib2 functions as an oncogene, and ectopic expression of Trib2 induces acute myelogenous leukemia following transduction into hematopoietic progenitor cells^12^. Additionally, Trib2 is a C/ebpα antagonist that mediates the E3 ligase COP1- dependent degradation of C/ebpα^13^,^14^. Trib2 also acts downstream of Wnt/TCF signal pathway in liver cancer cells to regulate YAP and C/ebpα^15^. Overexpression of Trib2 is also found in human lung cancers and contributes to tumorigenesis through downregulation of C/ebpα^16^. More recently, Trib2 is shown to be selectively expressed in the premegakaryocyte erythroid progenitors (preMegEs)^6^, and in a chromatin immunoprecipitation assay its promoter sequences were bound by Gata-2/Fog-1 in a population of sorted preMegEs^6^. These results prompted us to investigate the physiological role of Trib2 in hematopoietic lineage commitment and development.

Here, we report that knockout of *Trib2* in mice induced macrocytic anemia and increased vulnerability to hemolysis. We also observed an obvious decrease in erythroid progenitors, but not granulocytes or megakaryocytes, in *Trib2*^−/−^ mice. Both kinase domain and COP1-binding domain, that are essential to degrade *C/ebp*α, of Trib2 are required for its ability to promote erythropoiesis in the *Trib2*-/- bone marrow cells. However, our genetic study of *Trib2* and *c/ebp*α double knockout mice suggests that Trib2 could promote erythropoiesis via a C/ebpα-independent pathway. Promotion of hemoglobin production and increased expression of various globin mRNAs in zebrafish further confirmed the pro-erythropoietic function of Trib2.

## Results

### **Trib2** deficiency decreases erythrocytes and sensitizes mice to hemolytic stress

**Figure 1:**
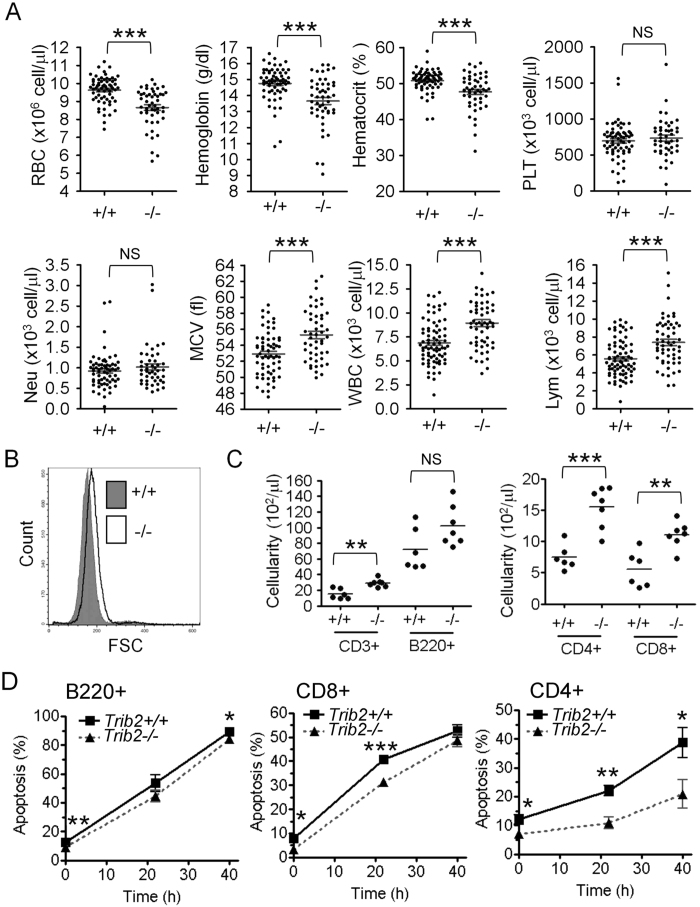
Trib2 deficiency confers macrocytic anemia on mice. (**A**) Complete blood count analyses of peripheral blood from *Trib2*^+/+^ (n = 63) and *Trib2*^−/−^ (n = 49) mice. 260 μL of blood was withdrawn into sodium citrate-containing tubes and subjected to complete blood counts using Abbott Cell-Dyn 3700. (**B**) Analysis of RBC cell volume of *Trib2*^+/+^ and *Trib2*^−/−^ mice by flow cytometry. 1 μL of blood was diluted with 200 μL of serum-PBS solution and analyzed by flow cytometry. (**C**) Numbers of CD3^+^, B220^+^, CD4^+^ and CD8^+^ cells in peripheral blood of *Trib2*^+/+^ (n = 6) and *Trib2*^−/−^ (n = 7) mice. WBCs were isolated from peripheral blood and characterized for specific surface markers by flow cytometry, as indicated. Absolute numbers of cells were calculated by multiplying the relative proportion of a particular cell population with the absolute number of WBCs. (**D**) Cytokine withdrawal-induced apoptosis in peripheral lymphocytes. WBCs were isolated from peripheral blood of *Trib2*^+/+^ and *Trib2*^−/−^ mice and cultured in cytokine-free medium *in vitro*. Apoptosis of B220+, CD8+, and CD4+ cells were analyzed by Annexin-V staining and specific surface markers by flow cytometry (n = 8 for each genotype). RBC, red blood cell; PLT, platelet; Neu, neutrophil; MCV, mean corpuscular volume; WBC, white blood cell; Lym, lymphocyte. The graphs show the mean and S.E.M., *P < 0.05 compares between the indicated groups. **P < 0.01 compares between the indicated groups. ***P < 0.005 compares between the indicated groups. NS: no significant difference.

To study the possible role of Trib2 in hematopoiesis *in vivo*, we employed a conventional homologous recombination gene targeting strategy using Svj 129 (R1) ES cells to generate *Trib2* knockout mice. The targeting strategy is described in Supplementary Figure S1, and unless otherwise indicated, all mice described below were offspring from the intercrosses between the N1 generations (see Supplementary Information). *Trib2*^−/−^ mice were viable and exhibited normal appearance in a C57BL6/129Svj mixed genetic background. Complete blood count analysis of peripheral blood from *Trib2*^+/+^ and *Trib2*^−/−^ mice revealed that *Trib2*^−/−^ mice had statistically significant decreased levels of RBC numbers, hemoglobin and hematocrit compared to *Trib2*^+/+^ mice (p < 0.005, Fig. 1A), but numbers of platelets and neutrophils were similar (Fig. 1A). In contrast, the mean corpuscular volume, white blood cell (WBC) count and lymphocyte count (Fig. 1A) were higher in *Trib2*^−/−^ mice. The increased mean corpuscular volume was consistent with the RBC volume, as assessed by flow cytometry (Fig. 1B), suggesting a phenotype of macrocytic anemia (Fig. 1A). Although the number of B220^+^ B cells was not significantly changed, the cell numbers of total CD3^+^, CD4^+^ and CD8^+^ T cells were significantly elevated in *Trib2*^−/−^ mice (Fig. 1C). This may be partly due to increase of resistance of T lymphocytes to cytokine deprivation induced apopotosis, as the percentage of apoptotic cells were lower in peripheral CD8+ and CD4+ T cells from *Trib2*^−/−^ mice than from *Trib2*^+/+^ mice in the *in vitro* culture (Fig. 1D). These results are in good accordance with our previous report on the role of Trib2 in promoting apoptosis during cytokine deprivation of hematopoietic cells^10^.

**Figure 2:**
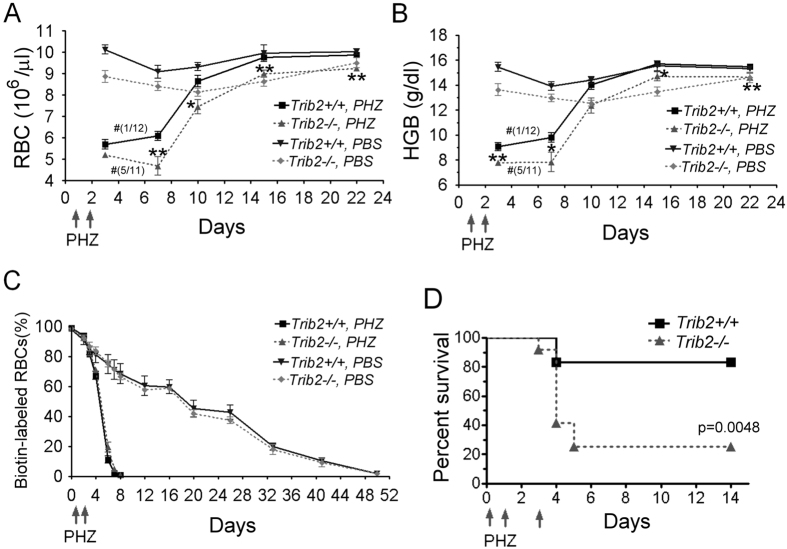
Trib2 deficiency sensitizes mice to hemolytic stress. (**A,B**) Hemolytic anemia was induced by two consecutive injections of PHZ on days 1 and 2 (50 mg/kg mice). Red blood cell counts (**A**) and hemoglobin (**B**) in the peripheral blood were measured by Abbott Cell Dyn 3700 at the indicated time points during recovery from anemia (n = 4 for the control group; n = 11~12 for PHZ-treated group). Symbol # indicates ratio of dead mice to total mice after the first bleeding. The graphs show the mean and S.E.M., *P < 0.05 compares between the indicated groups. **P < 0.01 compares between the indicated groups. (**C**) Decay profiles of biotin-labeled RBCs. Hemolytic anemia was induced as described in (**A,B**) (n = 4~6 for each group). (**D**) Kaplan–Meier survival curves of *Trib2*^+/+^ and *Trib2*^−/−^ mice after receiving three intraperitoneal injections of PHZ (50 mg/kg) at days 0, 1 and 3 (n = 12 for each genotype).

To further assess the importance of this mild defect in red blood cell development, we first challenged mice with a hemolytic agent — phenylhydrazine (PHZ, 50 μg/g body weight) — over two consecutive days. We withdrew blood at several time-points to analyze numbers of RBC and hemoglobin levels. Although PHZ treatment effectively reduced the RBC count (Fig. 2A) and hemoglobin concentration (Fig. 2B), the difference between *Trib2*^+/+^ and *Trib2*^−/−^ mice did not seem to be enlarged. This is consistent with observations that the *Trib2*^−/−^ and *Trib2*^+/+^ RBC have similar half-lives in the normal v.s. PHZ treatment condition and did not show any significant difference (Fig. 2C), confirming that the viable RBC number is not particularly sensitized to PHZ insult in the absence of Trib2. Furthermore, before and after hemolysis, total splenocyte and erythroblast numbers remained largely comparable between *Trib2*^+/+^ and *Trib2*^−/−^ mice, with hemolysis increasing the percentages of splenic erythroblasts in both *Trib2*^+/+^ and *Trib2*^−/−^ mice to a similar extent (Figure S2), suggesting that the emergent hematopoiesis in spleen remains equally effective without Trib2. However, we noticed that after the first blood withdrawal, more *Trib2*^−/−^ mice died (5 out of 11) compared to *Trib2*^+/+^ mice (1 out of 12) (see Fig. 2A,B). This observation prompted us to investigate the survival response of mice to the sublethal PHZ challenge by treating with three consecutive injections of PHZ, instead of two injections. In line with previous data, 9 out of 12 *Trib2*^−/−^ mice died after hemolytic challenge compared to 2 out of 12 *Trib2*^+/+^ mice (Fig. 2D). Therefore, although *Trib2* gene knockout only causes a mild reduction in RBC number at steady state in comparison to that of *Trib2*^+/+^ mice, *Trib2*^−/−^ mice are highly vulnerable to anemic insults, either by blood withdrawal or PHZ treatment.

### *Trib2* is preferentially expressed in hematopoietic progenitors, lymphoid and early erythroid lineages

**Figure 3:**
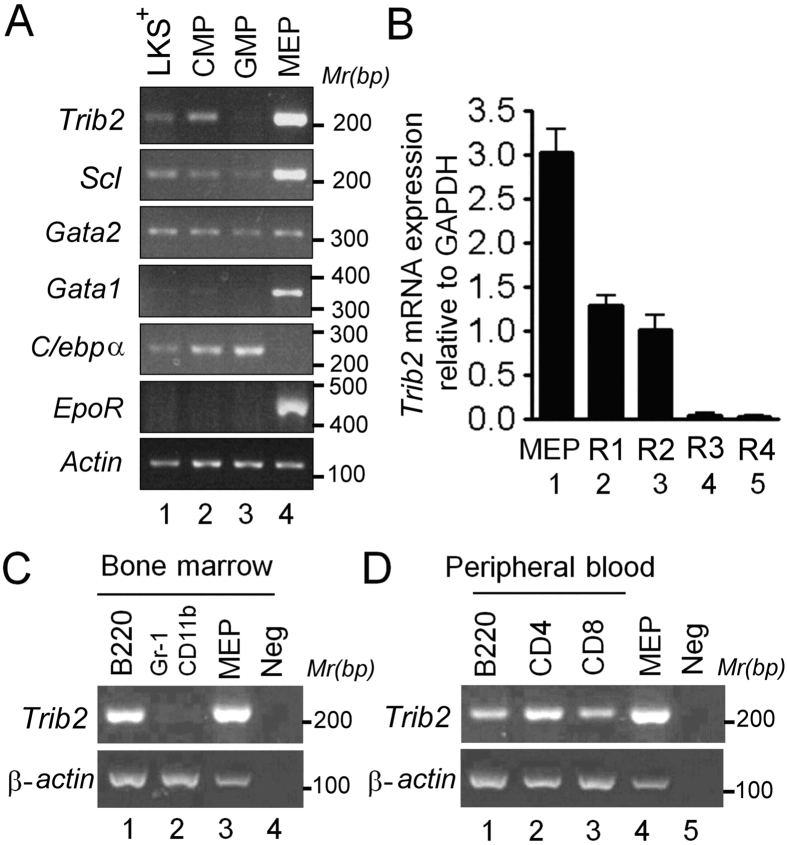
*Trib2* is preferentially expressed in hematopoietic progenitors of the erythroid lineage. (**A**) Semi-quantitative RT-PCR analysis for differential expression of mRNA of the *Trib2* gene and key regulatory transcription factors in various hematopoietic stem and progenitor cells. Bone marrow mononuclear cells were isolated from a femur of wild-type mice, and 5 × 10^7^ cells were subjected to FACS. See Supplementary Figure S3A for in-depth descriptions of LKS^+^, CMP, GMP and MEP. (**B**) Q-PCR of *Trib2* mRNA in MEPs and four developmental stages of *erythroblasts* in bone marrow. Total bone marrow cells were isolated and purified with antibodies for CD71 and Ter119. See Supplementary Figure S3B for definitions of R1, R2, R3 and R4, n = 4. (**C,D**) Semi-quantitative RT-PCR analysis for differential expression of *Trib2* in B220^+^ and Gr1^+^ CD11b^+^ cells in bone marrow (**C**), and for B220^+^, CD4^+^ and CD8^+^ cells in peripheral blood (**D**). LKS^+^, Lin^−^ c-kit^+^ ScaI^+^ cell; CMP, common myeloid progenitor; GMP, granulocyte/macrophage progenitor; MEP, megakaryocyte/erythrocyte progenitor; R1, proerythroblast; R2, basophilic erythroblast; R3, polychromatic erythroblast; R4, orthochromatic erythroblast.

Mancini *et al*. reported that *Trib2* was highly expressed in sorted *Trib2*^+/+^ bipotential megakaryocyte erythroid progenitors (Pre-MegE), but not in Fog-1 knockout Pre-MegE^6^. In our current study, we systematically monitored the expression levels of *Trib2* mRNA in the BM of both lineage-negative and lineage-positive populations. First, the lineage-negative (Lin^−^) population was enriched by MACS and then further subdivided into four separate populations consisting of LKS^+^ HSCs, CMPs, MEPs and GMPs according to methods described by Akashi *et al*.^17^ (Supplementary Figure S3A). The identities of the sorted progenitor populations were verified by expression of a set of marker genes, including *Scl* (for stem cells), *C/ebp*α (for myeloid lineages), and *Gata-1* and erythropoietin receptor (*EpoR*) (for MEPs and erythroid lineages) (Fig. 3A). *Trib2* mRNA was detectable in LKS^+^ HSCs and CMPs, and highly expressed in MEPs, but its expression was greatly diminished in GMPs, which was the opposite for the result for C/ebpα (Fig. 3A). Within the lineage-positive population, *Trib2* mRNA was detectable in erythroblasts, with lower expression in proerythroblasts (R1) and baso-erythroblasts (R2) (Fig. 3B). *Trib2* mRNA was undetectable in poly-erythroblasts (R3) and ortho-erythroblasts (R4) (Fig. 3B and Supplementary Figure S3B). In agreement with its lack of expression in GMPs, *Trib2* mRNA was also not detectable in mature Gr-1^+^ CD11b^+^ granulocytes (Fig. 3C, lane 2). However, *Trib2* mRNA could be continuously detected in lymphoid lineage cells, including B220^+^ B cells (Fig. 3C,D, lane 1) and peripheral CD4^+^ and CD8^+^ T cells (Fig. 3D, lanes 2 and 3). Yoshida *et al*.^18^ had reported a very similar expression profile of *Trib2* mRNA, however, only our data clearly and reproducibly showed that both *Trib2* and *C/ebp*α mRNA were detectable in LKS^+^ HSCs and CMPs population.

### Trib2 promotes erythroid lineage commitment of CMPs

**Figure 4:**
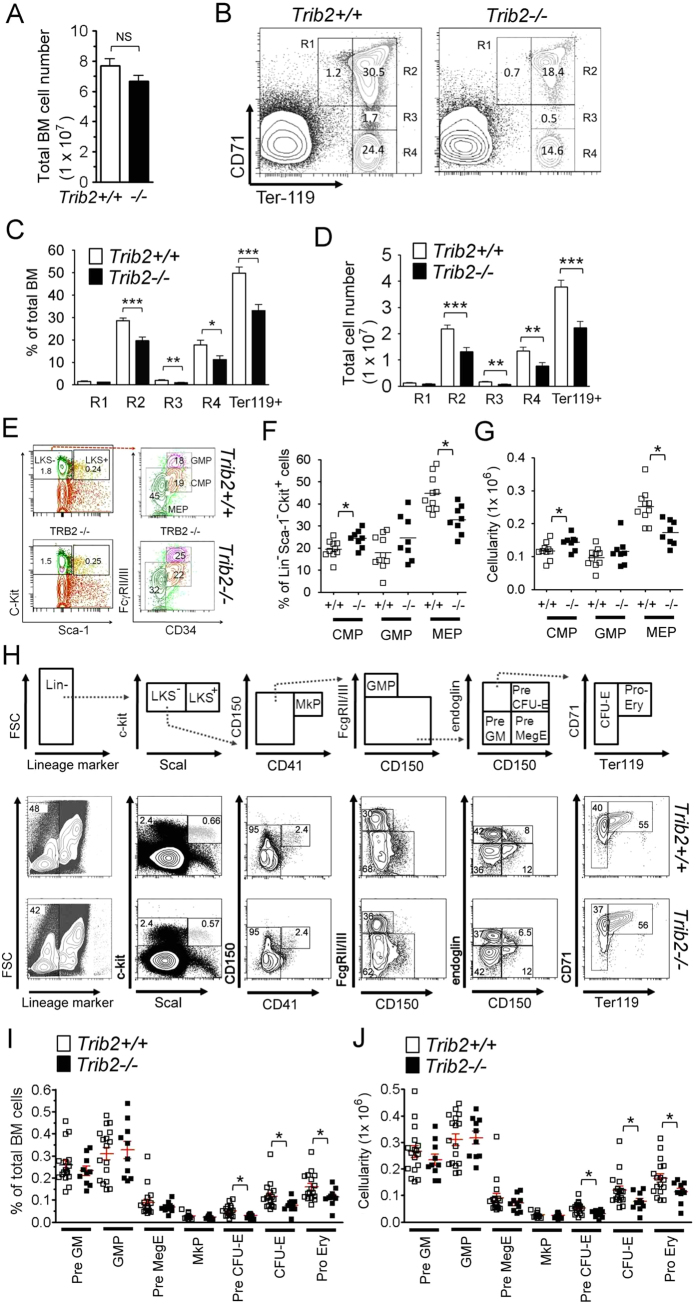
*Trib2* deletion reduces the erythrocyte lineage numbers *in vivo*. (**A**) Total bone marrow cellularity of *Trib2*^+/+^ and *Trib2*^−/−^ mice. (**B**) Reduced erythroblasts in *Trib2*^−/−^ bone marrow. Representative CD71/TER119 FACS profiles of bone marrow from *Trib2*^+/+^ and *Trib2*^−/−^ mice. (**C**) Statistical data analysis of (**B**), values were derived from CD71/TER119 FACS profiles and are presented as the mean ± SEM for 8–10 mice per genotype. (**D**) Absolute number of cells at individual erythroid differentiation stages (R1–R4). (**E,F**) Reduced MEP population in *Trib2*^−/−^ bone marrow. Total bone marrow cells were first subjected to gating of lineage markers (Lin) expression. The Lin^−^ fraction was then analyzed for Sca1 and c-kit expression (E, left panels). Lin^−^c-Kit^+^ ScaI^−^ (LKS^−^) cells were further analyzed for CD34 and FcγRII/III expression (E, right panels). The percentage of each population is shown. Quantification of each CMP, GMP and MEP population, together with statistical data, are shown in (**F**). (**G**) Absolute cell numbers of CMP, GMP and MEP in *Trib2*^+/+^ or *Trib2*^−/−^ BM. The graphs show the mean and S.E.M., *n* = 8–10 for each group. *P < 0.05 compares between the indicated groups. (**H**) Representative FACS profiles of *Trib2*^+/+^ and *Trib2*^−/−^ hematopoietic progenitors using the Pronk protocol^9^. The top panel shows the gating strategy and differential gates used to define various progenitor populations. (**I,J**) Percentages (**I**) and absolute cell numbers (**J**) of myelo‐erythroid progenitors of total BM cells were determined as described in (H) for *Trib2*^+/+^ (*n* = 17) and *Trib2*^−/−^ (*n* = 10) mice. The graphs show the mean and S.E.M., *P < 0.05 compares between the indicated groups.

The obvious reduction in RBC number in peripheral blood, despite there being no significant difference in steady-state and emergent erythroblast numbers in the spleen (Supplementary Figure S2), prompted us to analyze the early erythroid precursor and progenitor populations within BM. The *Trib2*^−/−^ mice constantly have similar cellularity in bone marrow like that of *Trib2*^+/+^ mice (Fig. 4A), and further flow cytometry analysis showed that within the lineage positive population of BM cells, the percentages and total cell numbers of total erythroblasts and most erythroblast subsets (mainly R2-R4) were all significantly decreased in *Trib2*^−/−^ mice (Fig. 4B–D). Since *Trib2* mRNA is expressed at the very early stages of hematopoiesis, we wanted to explore the possible defect elicited by *Trib2* knockout in myeloid progenitors. First, we analyzed the composition of LKS^+^ HSCs, CMPs, GMPs and MEPs using markers suggested by Akashi *et al*.^17^ and found that the cell number of MEPs was significantly decreased, whereas the cellularity of CMPs and GMPs was either slightly enhanced (CMPs) or not significantly altered (GMPs) in *Trib2*^−/−^ BM (Fig. 4E–G). A very similar erythroid phenotype was still observed in knockout mice generated from the intercrosses between the N5 generations (*Trib2*^+/−^ mice in C57BL6/129Svj mixed genetic background backcrossed to C57BL/6 for five generations, see supplemental Figure S4), albeit the viability of the N5 knockout mice was markedly reduced (Table S1 and see Discussion below). To gain a further insight into the effect on myeloid-erythroid progenitor sub-populations, we used markers CD150, Endoglin and CD41, as suggested by Pronk *et al*.^9^, to analyze the LKS^−^ population (Fig. 4H). Consistent with abovementioned observations, we did not find a significant difference in the total cell numbers (cellularity) of Pre-GM, GMP, Pre-MegE and MkP populations (defined in the upper panels of Fig. 4H) between *Trib2*^+/+^ and *Trib2*^−/−^ BM (Fig. 4H, lower panels, I, J). In contrast, total cell numbers (cellularity) of all erythroid lineages, including Pre CFU-E, CFU-E and Pro-Ery, showed significant reductions in *Trib2*^−/−^ BM (Fig. 4H, lower panels, I, J). These data accord well with our observation that *Trib2*^−/−^ mice have normal numbers of platelets in peripheral blood, and the defect is only restricted to the erythroid lineage (Fig. 1A).

**Figure 5:**
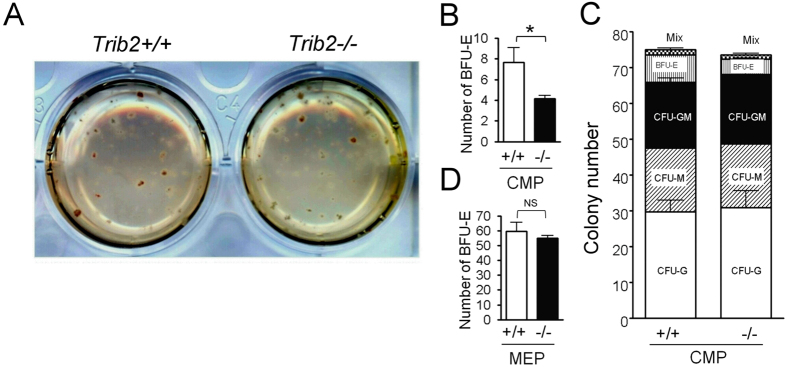
Deletion of Trib2 decreases BFU-E colonies of CMPs, but not MEPs. (**A–C**) CMPs were purified from *Trib2*^+/+^ and *Trib2*^−/−^ mice as described in Fig. 3A and subjected to an *in vitro* colony-forming unit (CFU) assay for differentiation into myeloid and erythroid lineages. (**A**) Representative images of CFU plates are shown. The sorted *Trib2*^+/+^ or *Trib2*^−/−^ CMPs were cultured in methylcellulose-based medium with recombinant cytokines and erythropoietin for 10 days. (**B**) The numbers of BFU-E derived from 200 sorted *Trib2*^+/+^ and *Trib2*^−/−^ CMPs were recorded from plates in (**A**) (n = 6). (**C**) Bar graphs show the number of each different type of colony observed in plates (**A**). (**D**) 200 sorted MEPs from *Trib2*^+/+^ and *Trib2*^−/−^ mice were cultured *in vitro* as described in (**A**), and the number of BFU-E was recorded (n = 4). The graphs show the mean and S.E.M. *P < 0.05 compares between the indicated groups. NS: no significant difference.

A colony-forming assay was used to re-confirm data obtained from flow cytometry. A sorted CMP population was subjected to colony assay and the identities of each colony were carefully characterized by high power microscopic images (Figure S5). Statistical analysis showed that the BFU-E of *Trib2*^−/−^ CMPs was significantly reduced (Fig. 5A,B), and that CFUs of myeloid lineage cells (including CFU-G, CFU-M, and CFU-GM) were not significantly different (Fig. 5C). Intriguingly, we found that sorted *Trib2*^−/−^ MEPs and *Trib2*^+/+^ MEPs produced comparable numbers of BFU-E (Fig. 5D), suggesting that Trib2 plays a more critical role in promoting erythroid development at the CMP stage and before cells enter the MEP stage.

### Pro-erythropoietic function of TRIB2 requires the kinase domain and COP1-binding domain

**Figure 6:**
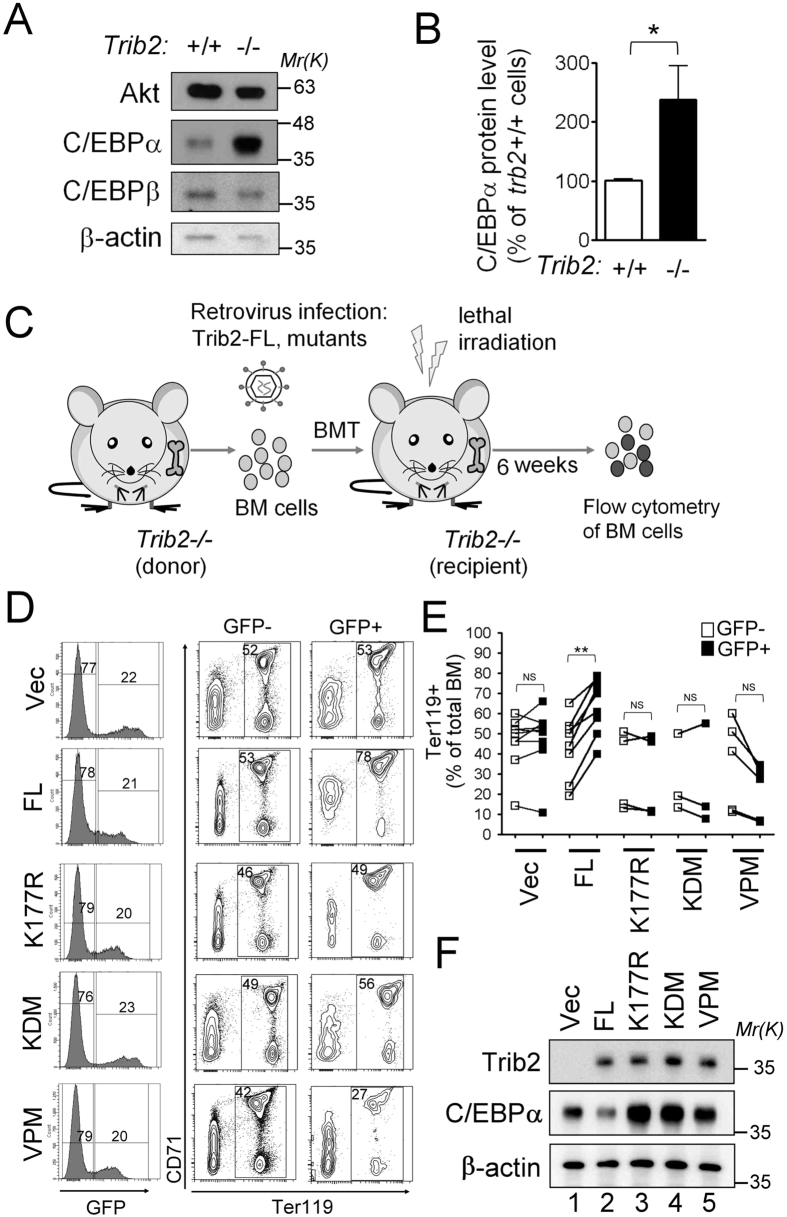
The kinase domain and COP1-binding region of Trib2 are essential for erythropoietic function and C/ebpα degradation. (**A**) Upregulation of C/ebpα in *Trib2*^−/−^ LKS^−^ cells. The LKS^−^ myeloid progenitors of *Trib2*^+/+^or *Trib2*^−/−^ bone marrow were isolated by FACS and subjected to Western blotting. Expression of endogenous C/ebpα was assessed with a specific antibody and a representative blot is shown. (**B**) Relative abundance of C/ebpα in (**A**) was determined by densitometry (n = 5). (**C**) Experimental scheme for Trib2 rescue and functional domain assessment. *Trib2*^−/−^ recipient mice were lethally irradiated and reconstituted with *Trib2*^−/−^BM cells transduced with control MSCV2.2-IRES-GFP vector or WT mouse Trib2 (FL) or Trib2 mutants (kinase-like domain (KD) mutants, K177R and KDM; COP1-binding site mutant, VPM). Flow cytometric analysis was performed at 6 weeks post BMT. (**D**) Analysis of the percentage of total erythroblasts in both GFP^−^ and GFP^+^ populations of each transplanted BM. Gene transduced BM cells were identified by the expression of the GFP surrogate marker (left panels). Both GFP^+^ and GFP^−^ fractions were subjected to erythroblast analysis with CD71 and Ter119 markers (right panels). Percentage numbers are given inside each panel. Results are representative of two independent experiments with 3–10 mice for each group. (**E**) Statistical data analysis of (**D**), values were derived from CD71/Ter119 FACS profiles and are presented as the mean ± SEM for 3–10 mice per group. Linked dots represent paired data that are derived from the same mouse. **P < 0.01 compares between the indicated groups. NS: no significant difference. (**F**) Effect of Trib2 mutants on C/EBP-α expression levels. Transduced *Trib2*^−/−^ BM cells were sorted for GFP expression 6 weeks after BMT, and assessed for the expression of Trib2 and C/EBP-α proteins by Western blot. β-actin is the protein loading control.

Since Trib2 overexpression in BM cells leads to degradation of C/ebpα^12^, we then investigated possible alterations in C/ebpα levels due to *Trib2* knockout. We observed a significant elevation of C/ebpα levels in *Trib2*^−/−^ LKS^−^ cells (Fig. 6A,B), suggesting that *Trib2* deletion could potentially inhibit erythrocyte development via elevation of C/ebpα levels. To further emphasize the importance of this C/ebpα-dependent regulation of erythrocyte development, we explored whether those structural domains essential for C/ebpα degradation — identified by Keeshan *et al*.^14^ — are important for Trib2-regulated erythroblast production. We constructed retroviral vectors containing either no cDNA, full-length Trib2 (FL), Trib2-K177R (K177R), Trib2-KDM (KDM) or Trib2-VPM (VPM). BM cells were isolated from *Trib2*^−/−^ mice and transplanted into a new *Trib2*^−/−^ mouse after being infected with a distinct Trib2-expressing retrovirus (Fig. 6C). Six weeks later, BM cells were harvested and both GFP^−^ and GFP^+^ cells were gated from total BM cells by flow cytometry. In this experiment, around 20% of BM cells were GFP+ for each individual retroviral infection (Fig. 6D, left panels) and both GFP^−^ and GFP^+^ populations were analyzed for erythroblast development with CD71 and Ter119 markers (Fig. 6D, right panels). As shown in Fig. 6E, only FL Trib2 could significantly elevate the percentages of erythroblasts in total transfected BM cells (i.e. GFP^+^) compared to that in un-infected BM cells (i.e. GFP^−^), but not other Trib2 mutants, suggesting that the Trib2 catalytic core motif and COP1-binding site are essential for promoting the erythropoiesis function in *Trib2*^−/−^ BM cells. The erythroblast rescue activity of Trib2 correlated very well with its ability to degrade C/EBPα (Fig. 6F), which supports a C/EBPα-dependent erythropoietic function of Trib2.

### Trib2 promotes erythrocyte development via a C/ebpα-independent mechanism by genetic approach

**Figure 7:**
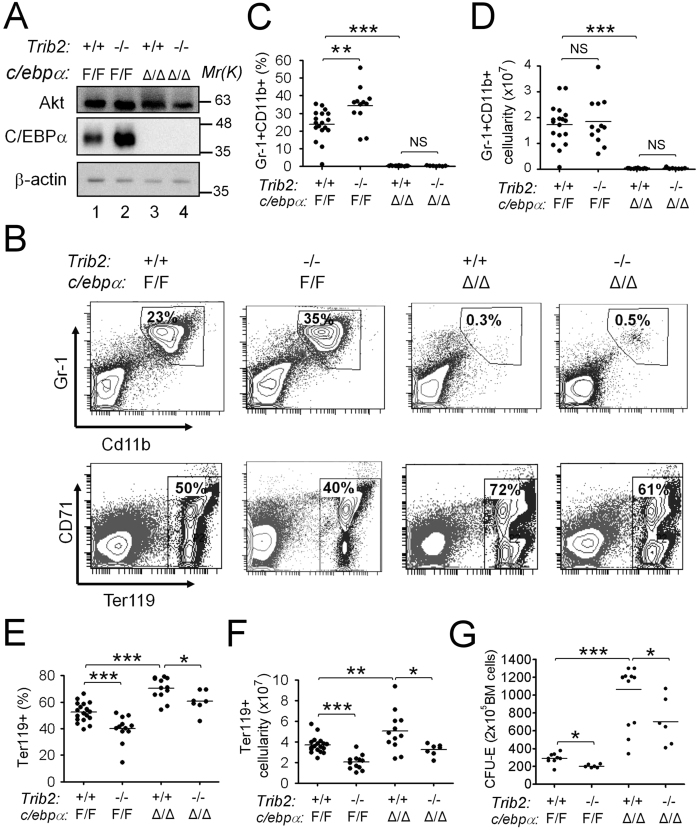
Genetic study revealed a C/ebpα-independent erythropoietic function of Trib2. (**A**) Absence of C/ebpα expression in Mx1-Cre+; *C/ebp*α^*F/F*^ mice after poly I:C treatment. *Trib2*^+/+^; *C/ebp*α^*F/F*^, *Trib2*^−/−^; *C/ebp*α^*F/F*^, *Trib2*^+/+^; Mx1-Cre+; *C/ebp*α^*F/ F*^, and *Trib2*^−/−^; Mx1-Cre+; *C/ebp*α^*F/F*^ mice were all injected with poly I:C. Three weeks later, total bone marrow cells were isolated and subjected to Western blotting with specific antibodies as indicated. (**B–F**) Deletion of *Trib2* reduces erythroblast differentiation in *C/ebp*α^*F/F*^ and *C/ebp*α^Δ/Δ^ genetic backgrounds. (**B**) A representative FACS profile for each mouse group (as indicated on top of panels). The percentage of CD11b^+^ Gr-1^+^ cells (upper panels) or Ter119^+^ cells (lower panels) is indicated in each panel. Statistical analysis of granulocyte data (B, upper panels) is shown in (**C,D**), and that of erythroblasts (B, lower panels) is shown in (**E,F**). The graphs show the mean and S.E.M., *n* = 7–18 for each group. *P < 0.05 compares between the indicated groups. **P < 0.01 compares between the indicated groups. ***P < 0.005 compares between the indicated groups. NS: no significant difference. (**G**) Colony forming unit assay of erythrocytes (CFU-E) was performed for total bone marrow cells isolated from Trib2 and C/ebpα knockout mice, as indicated. The graph shows the mean and S.E.M., *n* = 6–11 for each group. *P < 0.05 compares between the indicated groups.

Although the above experiments strongly suggest the importance of a C/EBPα-dependent mechanism in Trib2-regulated erythropoiesis, it does not formally reject the possibility of the role of a C/EBPα-independent mechanism, especially in most of erythroid cells Trib2 is highly expressed without the presence of C/ebpα (Fig. 3 and ref. 18). To investigate the possible interplay between Trib2 and C/ebpα in erythroid development. we decided to take a genetic approach. We generated *C/ebp*α and *Trib2* double knockout (*C/ebp*α^Δ/Δ^*; Trib2*^−/−^) mice by crossing *Mx1-cre*, *C/ebp*α^F/F^ and *Trib2*^−/−^ mice, and we ablated the *C/ebp*α gene in hematopoietic cells by injecting poly I:C into young adult mice. Three weeks after poly I:C injection, total BM cells were purified and analyzed for C/ebpα expression by Western blotting. *Trib2* knockout resulted in higher C/ebpα levels than controls (Fig. 7A, compare lanes 1 and 2), but C/ebpα was almost undetectable when *C/ebp*α was ablated. Consistent with that observed in Fig. 4A–D, in this double crossing experiment, single deletion of *Trib2* resulted into the decrease of the erythroblasts in BM both in percentages as well as in absolute cell numbers (Fig. 7B,E,F, compare left two columns). Notably, although the granulocyte percentage was increased in mice with single deletion of the*Trib2* gene, (Fig. 7B,C, left two columns), the total granulocyte cell number (cellularity) in these *Trib2* knockout mice remained very similar to that of the control mice (Fig. 7D). On the other hand, in *C/ebp*α-single deleted mice, granulocyte levels (both percentages and total cell numbers) were markedly reduced (Fig. 7B–D), whereas their erythroblast levels were significantly increased compared to control mice (50% in *Trib2*^+/+^; *C/ebp*α^F/F^ v.s. 72% in *Trib2*^+/+^; *C/ebp*α^Δ/Δ^ mice, Fig. 7B). Significantly, in the absence of *C/ebp*α, deletion of *Trib2* still resulted into the decrease of erythroblasts in the BM (Fig. 7B, 61% *in Trib2*^−/−^; *C/ebp*α^Δ/Δ^ mice v.s. 72% in Trib2^+/+^; *C/ebp*α^Δ/Δ^ mice; Fig. 7E,F). The reduction in erythroid precursor numbers was also verified by colony-forming assays in which combined deletion of *Trib2* and *C/ebp*α further reduced CFU-E numbers, compared to deletion of *C/ebp*α alone (Fig. 7G, compare ~1100 colonies in *Trib2*^+/+^; *C/ebp*α^Δ/Δ^ mice to ~700 colonies in *Trib2*^−/−^; *C/ebp*α^Δ/Δ^ mice). Together, these results strongly suggest that, in the physiological condition, Trib2 promotes erythrocyte development via a C/ebpα-independent mechanism.

### Induction of erythropoiesis in h*Trib2* mRNA–injected zebrafish embryos

**Figure 8:**
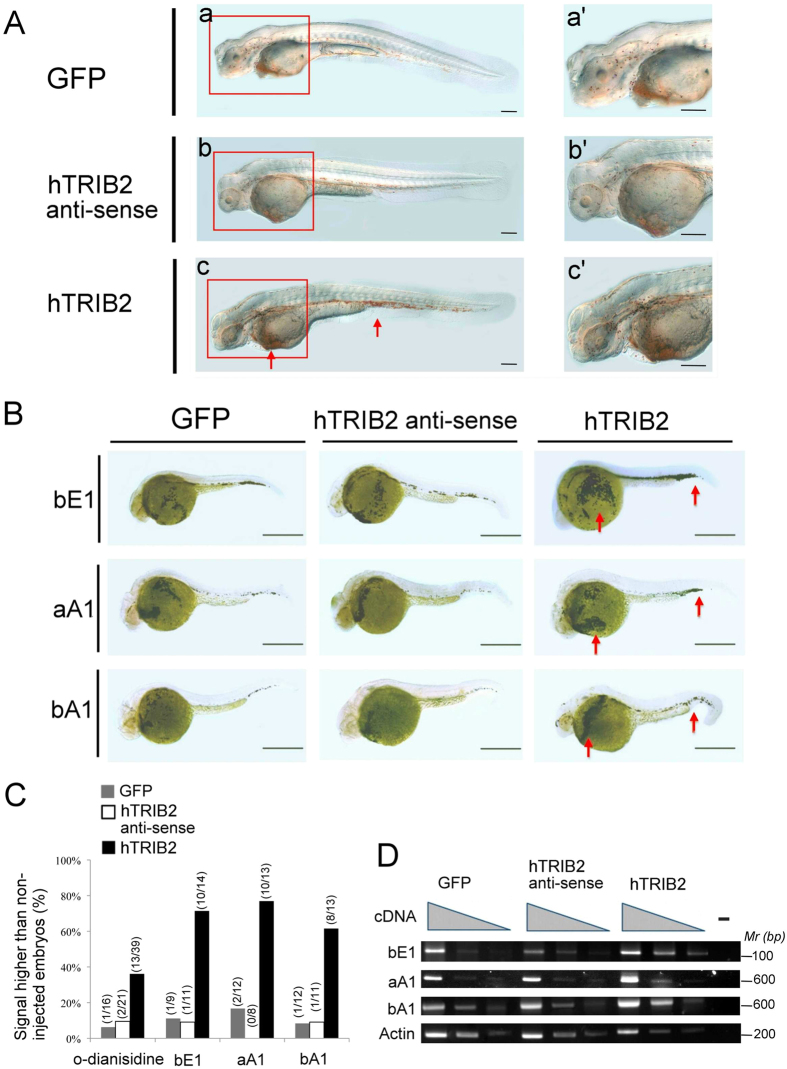
Elevated hemoglobin production and increased expression of erythroid-specific genes in zebrafish embryos injected with h*TRIB2* mRNA. (**A**) *o*-dianisidine staining of embryos injected with GFP mRNA (a, a′), h*TRIB2* anti-sense mRNA (b, b′) or h*TRIB2* mRNA (c, c′) was performed at 48 hpf. Hemoglobin staining (red arrows) was significantly increased in *hTRIB2* mRNA–injected embryos. (**B**) Whole-mount *in situ* hybridizations with antisense β*e1-globin* (bE1), α*A1-globin* (aA1) and β*A1-globin* (bA1). The images weretaken from a lateral view, with anterior to the left and dorsal to the top. Embryos were stained under the same conditions and for the same period of time to ensure comparable sensitivity. Multiple embryos were injected and analyzed, and statistical results are shown in (**C**). The number of embryos with a positive signal divided by the number of embryos injected is shown at the top of each bar. Two independent experiments were performed, and one representative result is shown. (**D**) Semi-quantitative RT-PCR for differential expression of zebrafish β*e1-globin* (bE1), α*A1-globin* (aA1) and β*A1-globin* (bA1), and β*-actin* in GFP mRNA, hTRIB2 anti-sense mRNA and h*TRIB2* mRNA–injected embryos at 22 hpf. Similar results were obtained from three experiments using separate embryos injected with GFP mRNA, h*TRIB2* anti-sense mRNA and h*TRIB2* mRNA. Scale bars: 100 μm in (**A,B**).

Finally, we investigated whether Trib2 overexpression could promote erythropoiesis in zebrafish. For these studies, we selected zebrafish (which has an erythropoietic system very similar to that in mammals^19^,^20^) to examine the pro-erythropoietic effects of Trib2. *Human Trib2* mRNA was injected into zebrafish embryos at the 1- to 2-cell stage, and 48 h later the presence of hemoglobin was detected by o-dianisidine staining (Fig. 8A). Embryos injected with *hTrib2* mRNA at 48 hours post-fertilization (hpf) displayed a significant increase in hemoglobin compared with embryos injected with control GFP mRNA and *hTrib2* antisense mRNA, of which neither of these latter exhibited a significant change in hemoglobin compared to non-injected controls. Although human Trib2 has only 78% amino acid sequence identity with zebrafish Trib2, our results showed that this level of identity was high enough to allow human Trib2 to function in zebrafish, but its nucleotide identity (72.9%) is too low to create an antisense suppressive effect against the zebrafish *Trib2* mRNA when human *Trib2* antisense RNA was expressed. We also analyzed the effect of *hTrib2* mRNA injection on expression of erythroid-specific genes — *bE1*-globin, *aA1*-globin and *bA1*-globin — by whole-mount *in situ* hybridization. Expression of the examined erythroid-specific genes increased significantly in *hTrib2* mRNA–injected embryos at 28 hpf (Fig. 8B). Significantly higher percentages of embryos injected with *Trib2* mRNA showed increased staining by *in situ* hybridization, but embryos injected with GFP mRNA and anti-sense RNA did not show the same effect (Fig. 8C). The induction of erythroid-specific genes in *hTrib2* mRNA–injected embryos was also confirmed by semi-quantitative PCR (Fig. 8D). Altogether, our results suggest that ectopic expression of Trib2 positively regulates erythropoiesis *in vivo*.

## Discussion

The Tribbles protein regulates cell proliferation, migration, and morphogenesis during Drosophila embryo development^21^,^22^, but the functions of the mammalian Tribbles family *in vivo* remain largely unknown. To investigate the effects of Trib2 on mammalian physiology, we generated Trib2-deficient mice using a conventional gene targeting approach. The *Trib2*^−/−^ mice displayed a normal appearance, but abnormal complete blood count analysis, including reductions in RBCs and hemoglobin levels in peripheral blood (Fig. 1) and elevated WBC (mainly CD4^+^ T lymphocytes; Fig. 1) in peripheral blood. Further flow cytometric analyses revealed a defect in early development of erythroid lineage, including reduction of erythroblasts, Pre-CFU-E, CFU-E and Pre-Ery (Fig. 4). A very similar erythroid phenotype was also observed in knockout mice generated from the intercrosses between the N5 generations (backcrossed to C57BL/6 for five generations, see supplemental Figure S4). However, we noticed that starting from the P2 stage the percentage of Trib2^−/−^ mice from the intercrosses between mice from the N5 generations was significantly less than that would be expected from the Mendelian frequency (supplemental Table S1), suggesting that Trib2 deficiency would result into a more severe phenotype with perinatal lethality in the C57BL/6 genetic background. Whether the perinatal lethality phenotype is mainly due to the defect in the erythroid development remains to be determined.

The elevated cell numbers and resistance to cytokine depletion-induced apoptosis of *Trib2*^−/−^ CD4^+^ T cells are consistent with our previous observations that Trib2 is involved in promoting IL-2 withdrawal-induced T cell apoptosis^10^. More interestingly, although the hematological indexes of *Trib2*^−/−^ mice only altered slightly, *Trib2*^−/−^ mice were very sensitive to sublethal doses of a hemolytic agent or anemic stress. One possibility is that the mice would die when RBC indexes dropped below an unknown threshold, and that in our experiments the indexes of *Trib2*^−/−^ mice went just below this threshold after treatment with a higher dose of hemolytic agent or bleeding, but this did not occur in *Trib2*^+/+^ mice. Alternatively, since the oxidative stress generated after hemolysis of RBC is extremely high, the *Trib2*^−/−^ mice may not have produced enough reducing power inside their blood and vessels to protect them, and so died faster than their *Trib2*^+/+^ littermates. Finally, hemolysis of Trib2-deficient RBC may generate much higher oxidative stress than that in *Trib2*^+/+^ mice so that the former are more sensitive to hemolytic agents. This phenomenon is very intriguing and may have important clinical relevancy, and is very much worthy of further extensive investigation.

Recently, Keeshan *et al*.^11^ showed that Trib2 regulates the fate of myeloid precursor cells by modulating the level of the critical myeloid differentiation transcriptional factor, C/ebpα, and found that ectopic Trib2 overexpression in myeloid progenitors resulted in development of acute myeloid leukemia in mice^12^. It remains to be investigated whether *Trib2*^−/−^ progenitors in BM will be refractory to myeloid leukemia development owing to the tumor suppressive function of accumulated C/ebpα. In our *Trib2*^−/−^ mice, *Trib2* ablation resulted in accumulation of endogenous C/ebpα and decreased RBC production. Moreover, ectopic expression of Trib2 in *Trib2*^−/−^ BM cells led to decreased C/ebpα levels and promoted erythroblast production (Fig. 6). Through further analysis with Trib2 mutant proteins, our data suggested that the kinase motif and COP1-binding region of Trib2 are important for C/ebpα degradation and for promotion of erythrocyte production. Therefore, Trib2 seems to have a C/ebpα-dependent erythrocyte promotion function. However, through a loss-of-function genetic approach it was concluded that Trib2 promotes *in vivo* erythrocyte development via a C/ebpα-independent mechanism (Fig. 7). The discrepancy between results of genetic study and that of the ectopic expression experiments could be due to the fact that in neither granulocytic nor erythroid progenitors the endogenous C/ebpα and Trib2 proteins are co-expressed. Therefore, when *Trib2* or *C/ebp*α gene is knocked out, the result is the sum of two independent events. However, during ectopic expression by gene transfection, Trib2 could be artificially expressed in granulocytic cells, where C/ebpα is highly expressed and is sensitive to Trib2-mediated degradation, and resulted in a C/ebpα protein level-dependent erythrocyte production.

Due to the sickness of *Trib2* and *C/ebp*α double knockout mice, we were unable to carry out a similar gene delivery experiment as in Fig. 6 for the *Trib2* and *C/ebp*α double knockout mice before the animals became too sick and moribund. Thus, whether these functional domains of Trib2 are also important for C/ebpα-independent erythrocyte promotion and what is the C/ebpα-independent Trib2-dependent erythropoeisis pathway remains to be investigated. A preliminary comparison of mRNA expression levels of *Gata-2, Fog-1, Pu.1* and *Gata-1* genes in *C/ebp*α^Δ/Δ^ and *C/ebp*α^Δ/Δ^; *Trib2*^−/−^ CMPs (data not shown) ruled out involvement of these genes downstream of Trib2 (unpublished data), but this issue certainly warrants further investigation.

In this study, due to the limitation in the availability of erythroblast-specific genetic variant markers, it is not possible for us to directly address whether the erythrocyte lineage defect observed in the *trib2*^−/−^ mice is cell intrinsic. The results shown in Fig. 6 indicate that, in the absence of Trib2 *in the* BM niche of the recipient mice, re-expression of wt Trib2 in *trib2*^−/−^ BM rescued erythroblast production in *trib2*^−/−^ mice, suggesting that the erythrocyte lineage defect of the *trib2*^−/−^ mice is likely cell intrinsic. But a role of BM niche in Trib2-regulated erythrocyte development cannot be excluded. More experiments would be required to address this important issue.

Zebrafish orthologs of many key transcriptional factors necessary for mammalian hematopoietic development have been identified, suggesting that a conserved genetic program regulates vertebrate hematopoiesis^23^. Data from our current study also suggest that the pro-erythropoiesis function of Trib2 is evolutionarily-conserved. The average homologies of nucleotide and amino acid sequences between human Trib2 and zebrafish Trib2 are 72.9% and 78.0%, respectively, but they appear to be functionally equivalent, and human Trib2 could induce RBC production in fish (Fig. 8A). Therefore, the zebrafish model may be useful in assessing the biological functions of various mutants and recombinants of human Trib2.

## Methods

### Animals and reagents

This study was conducted in strict accordance with recommendations in the Guide for the Care and Use of Laboratory Animals of the National Institutes of Health, USA. The protocol was approved by the Institutional Animal Care and Utilization Committee of Academia Sinica (protocol number: RMiIBMYJ2007066). Mice were euthanized with carbon dioxide, and all efforts were made to minimize suffering. *C/ebp*α^*F/F*^ conditional mice and Mx1- and E2A-cre mice were purchased from JAX lab (Bar Harbor, ME). The gene targeting strategy and generation of *Trib2*^−/−^ mice was based on conventional homologous recombination techniques and is described in detail in the Supplementary Information (see *Scientific Reports* website). Unless otherwise indicated, all mice analyzed in this study were offspring from the intercrosses between the N1 generations. (see Supplementary Information). The *Trib2*^−/−^ mice were maintained in a specific pathogen-free animal facility. Phenylhydrazine (PHZ), propidium iodide, and o-dianisidine were purchased from Sigma (St., Louis, MO). Poly I:C was purchased from InvivoGen (San Diego, CA).

### Cytokine withdrawal-induced apoptosis in peripheral lymphocytes

To prepare white blood cells, peripheral blood from *Trib2*^+/+^ and *Trib2*^−/−^ mice was treated twice with *ACK lysis* buffer (0.15 M NH_4_Cl, 10 mM KHCO_3_, 0.1 mM Na_2_EDTA) to eliminate RBCs. White blood cells were cultured in RPMI medium (20% FBS) in the absence of survival cytokines. Apoptosis of B220+, CD8+, and CD4+ cells were analyzed at indicated time points by Annexin-V (BioVision, CA) staining and specific surface markers by flow cytometry.

### Flow cytometry analyses and sorting of hematopoietic progenitors

Isolated single-cell suspensions of splenocytes or BM cells were stained with propidium iodide to monitor cell viability, and splenocytes were also pre-incubated with an FcγR-blocking antibody (clone 2.4G2) to block non-specific binding of antibodies to cell-surface FcγR. The cells were then stained with fluorochrome-conjugated specific antibodies as indicated for each experiment. All flow cytometric experiments were analyzed using a FACSCanto flow cytometer (BD Biosciences).

CMPs and MEPs were purified according to a previous published protocol^17^. In brief, lineage-negative cells in the IL-7 Rα^−^ population were first enriched from BM with a Magnetic Activated Cell Sorting (MACS) system (Miltenyi Biotec GmbH), followed by staining for c-Kit, Sca-1, FcγRII/III and CD34, and sorted using a FACSAria cell sorter (BD Biosciences). The Lin^−^c-Kit^+^Sca-1^−^CD34^+^FcγRII/III^int^ population was defined as CMPs, and the Lin^−^c-Kit^+^Sca-1^−^CD34^−^FcγRII/III^low^ population was defined as MEPs. Purified CMPs or MEPs were seeded in methylcellulose cultures and verified by *in vitro* colony assays^17^. To analyze hematopoietic stem and myeloid progenitor cells in detail, a published protocol^9^ was followed. In brief, unfractionated bone marrow cells were stained with unconjugated rat antibodies against mouse IL-7 Rα, CD4, CD8, B220, Gr1 and CD11b, and visualized with PE-conjugated goat anti-rat IgG antibody. Lineage-stained cells were subsequently stained with antibodies against Sca1, c-kit, FcγRII/III (FcgR), CD41 (Itga2b), CD150 (Slamf1), Endoglin (Eng/CD105), CD71 and Ter119. Propidium iodide was used to exclude dead cells. Cells were analyzed with flow cytometry. The sources of antibodies are listed in detail as Supplementary Information (see *Scientific Reports* website).

### Western blot analysis

Polyclonal anti-mouse Trib2 was generated as described^10^. Polyclonal anti-mouse C/EBPα and anti-mouse C/EBPβ were obtained from Santa Cruz Biotechnology (*Santa Cruz*, CA). Polyclonal anti-mouse Akt were obtained from Cell Signaling Technology Inc. Anti β-actin was purchased from Sigma. For Western blot analysis, cells to be analyzed were lysed in a NP-40 lysis buffer^10^, and 50 μg of cell lysate proteins were analyzed by Western blotting using antibodies as indicated in each figure.

### Construction of retroviral vectors for Trib2 mutants

A 1032 bp fragment encoding the entire mouse Trib2 cDNA was subcloned into the BglII and XhoI sites of the MSCV2.2-IRES-GFP vector. Site-directed mutagenesis of the catalytic domain (i.e. Trib2-K177R and Trib2-KDM mutants) and the COP1-binding site (i.e. Trib2-VPM mutant) of Trib2 was performed using the QuikChange Lightning Site-Directed Mutagenesis kit (Agilent) according to the manufacturer’s protocol. The first lysine of the Trib2 “LRDLKLRK” putative ser/thr kinase catalytic loop is mutated to arginine in Trib2-K177R (K177R), the Trib2 putative loop sequence is changed to the canonical ser/thr kinase sequence “LRDLKPEN” in Trib2-KDM (KDM) and the COP1-binding site “DQLVP” is altered to “AQLAA” in Trib2-VPM (VPM)^14^. Infectious but replication-incompetent recombinant retroviruses expressing Trib2, Trib2-K177R, Trib2-KDM and Trib2-VPM were generated in the PhoenixEco packaging cell line.

### RBC half-life determination

To determine the life span of RBC, mice were biotinylated *in vivo* with *N*-hydroxysuccinimido-biotin (NHS-biotin) (Sigma) as described previously^24^. Briefly, NHS-biotin was dissolved in DMSO and diluted 1:10 with PBS to a final concentration of 6 mg/ml, and 200 μl of the final solution was injected into the tail vein. 50 μl of blood from biotinylated mice was taken at the indicated time points and washed with 2 ml of PBS supplemented with 2% fetal bovine serum and 1 mM EDTA to remove biotinylated plasma proteins. Samples containing 2 μl of blood cells were stained with 0.5 μg of streptavidin-APC-Cy7 (Biolegend) for 30 min. The percentage of labeled RBC was determined by flow cytometry, and the disappearance of the RBC over time was used to determine the erythrocyte life span for each mouse.

### Retrovirus infection of BM

BM cells were collected from 8-week-old *Trib2*^−/−^ mice 4 days after intravenous administration of 5-FU (150 mg/kg, Sigma) and transduced with retrovirus *ex vivo* in the presence of IL-3, IL-6, and SCF^14^. Retroviral supernatants with equal titers were used to produce similar transduction efficiencies. Cells (1 × 10^6^) were then injected intravenously into lethally irradiated (950 rads) *Trib2*^−/−^ recipients. Chimeric mice were maintained on antibiotics for 2 weeks and assessed for engraftment 6 weeks post-BM transplantation (BMT), using GFP as a marker.

### *In vitro* colony-forming assays

For detection of CFU-E colonies, aliquots containing 2 × 10^5^ BM cells resuspended in 0.7 mL of erythropoietin-containing methylcellulose culture (MethoCult M3334; StemCell Technologies, Vancouver, Canada) were plated into 12-well plates according to the manufacturer’s protocol, and CFU-E colonies were counted after 2 days of culture. To detect BFU-E, CFU-GM, CFU-M, CFU-G and mixed CFU colonies, aliquots containing 200 CMPs or MEPs resuspended in 0.7 mL of multiple cytokine-containing methylcellulose culture (MethoCult M3434; StemCell Technologies) were plated into 12-well plates, and the colonies were counted after 10 days of culture^17^,^25^.

### Poly I:C–induced excision of *C/ebp*α^*F/F*^ in *Trib2* ^−/−^ mice

To remove the floxed allele of *C/ebp*α (*C/ebp*α^F^), Mx1-Cre+; *C/ebp*α^F/F^; *Trib2*^+/+^ or Mx1-Cre+; *C/ebp*α^F/F^; *Trib2*^−/−^ mice were injected with poly I:C (450 μg per mouse every other day for a total of seven injections)^25^. Three weeks after poly I:C treatment, excision of *C/ebp*α^F^ alleles was confirmed by Western blotting of C/ebpα protein in BM cells.

### Microinjection of *hTrib2 mRNA* in zebrafish embryos

Zebrafish were raised and maintained under standard conditions^26^. Embryos were incubated at 28 °C, and developmental stages were determined according to the descriptions in the Zebrafish Book^26^. Capped GFP and human *Trib2 (hTrib2)* sense RNA was synthesized with the mMESSAGE mMACHINE T7 Ultra kit (Ambion, Austin, TX) from linearized pT7TS plasmids containing the entire coding region of *hTrib2* cDNA (which shared 72.9% peptide *sequence identity* with zebrafish *Trib2* cDNA). Aliquots containing 30 pg of GFP and *hTrib2* sense RNA or *hTrib2* antisense RNA were injected into each embryo at the 1- to 2-cell stage. The RNA–injected embryos were dechorionated and fixed for *o*-dianisidine staining and whole-mount *in situ* hybridizations with antisense β*e1-globin* (bE1), α*A1-globin* (aA1) and β*A1-globin* (bA1) as described^27^.

### Semi-quantitative RT-PCR and real-time quantitative PCR analyses

For semi-quantitative analysis, 200 ng of RNA was subjected to reverse transcription (RT) into cDNA with oligo dT and SuperScript III reverse transcriptase (Invitrogen) in a total reaction volume of 20 μL. Gene expression in mice was analyzed by amplifying 3 μL of reverse- transcribed cDNA product from hematopoietic cells with specific primers. Gene expression in zebrafish embryos was determined by PCR amplification performed with zebrafish *bA1*, *aA1*, *bE1* and *β-actin* primers^27^. Real-time quantitative PCR was performed using the ABI 7500 Real Time PCR System (Applied Biosystems, Foster, CA).

### Primer information

The nucleotide sequences of primers used for mutagenesis, genotyping and semi-quantitative RT-PCR are provided as supplementary information in Scientific reports website.

### Statistical analysis

Statistical analysis was performed by using GraphPad Prism 4.0 software (San Diego, CA). Animal survival studies were plotted on Kaplan–Meier curves and analyzed using the log rank test. An unpaired Student’s *t-*test was used to determine significance of data unless otherwise stated. All error bars represent standard error of the mean (S.E.M.) unless otherwise indicated.

## Additional Information

**How to cite this article**: Lin, K.-R. *et al*. Murine tribbles homolog 2 deficiency affects erythroid progenitor development and confers macrocytic anemia on mice. *Sci. Rep.* **6**, 31444; doi: 10.1038/srep31444 (2016).

## Supplementary Material

Supplementary Information
