# Supplementary material for: Murine tribbles homolog 2 deficiency affects erythroid progenitor development and confers macrocytic anemia on mice
Source: Sci Rep. 2016 Aug 23;6:31444. doi: 10.1038/srep31444 (PMC4994003; doi:10.1038/srep31444)
Supplement: Supplementary Information [file srep31444-s1.pdf]

Murine tribbles homolog 2 deficiency affects erythroid progenitor development and confers macrocytic anemia on mice

Kou-Ray Lin,[1,8] Hsin-Fang Yang-Yen,[2] Huang-Wei Lien[3], Wei-Hao Liao,[4] Chang-Jen Huang,[4] Liang-In Lin,[6] Chung-Leung Li,[5] and Jeffrey Jong-Young Yen[1,7,8*]

[1]Institute of Biomedical Sciences, [2]Institute of Molecular Biology, [3]Institute of Cellular and Organismic Biology, [4]Institute of Biological Chemistry, and [5]Genomic Research Center, Academia Sinica, Taipei, Taiwan; [6]Department of Clinical Laboratory Science and Medical Biotechnology, and [7]Institute of Molecular Medicine, School of Medicine, National Taiwan University, Taipei, Taiwan; and [8]Taiwan Mouse Clinic, Academia Sinica, Taipei, Taiwan

***Correspondence** should be sent to Jeffrey J.Y. Yen, Institute of Biomedical Sciences, Academia Sinica, No. 128, Sec. 2, Yen-Jiou-Yuan Rd., Taipei 11529, Taiwan; e-mail: bmjyen@ibms.sinica.edu.tw; Phone: 886-2-26523077; FAX 886-2-26523081.

Supplementary Methods

Generation of Trib2 knockout mice and genotyping

The gene targeting strategy for *Trib2* is illustrated in Supplementary Figure S1A, wherein exons 1 and 2 of *Trib2* were replaced by a LacZ/Neo cassette and did not encode Trib2 protein. Genomic fragments harboring the entire *Trib2* locus were isolated from a 129/Svj mouse genomic library and used to construct the targeting vector (Fig. S1A-b). R1 ES cell targeting was generated by conventional gene targeting technology. Southern blotting using 5' and 3' probes, as indicated in Figure S1, was carried out to select clones that had undergone homologous recombination at the *Trib2* locus (Fig. S1A-c). Two ES cell clones harboring the deleted allele "-" (#177 and #321) were further microinjected into C57BL/6 blastocysts to generate chimeric mice and then to generate $Trib2^{+/-}$ mice. To generate neo-resistance cassette-free *Trib2* knockout mice, $Trib2^{+/-}$ female mice carrying the neo^r gene were crossed to EIIa Cre transgenic (Cre^{tg}) male mice (in C57BL/6 background) to remove the neo^r gene (Fig. S1-d). This deletion leaves only one loxP site in intron 2. The neo-excised/Cre^{tg} heterozygous mice were then backcrossed to C57BL/6 mice for one generation to segregate the Cre transgene. The resultant neo-excised/Cre^{tg}-segregated $Trib2^{+/-}$ heterozygous mice (designated as the N1 generation) were intercrossed for experimental use and maintained in a 129/C57BL/6 mixed genetic background. Unless otherwise indicated, all mice analyzed in this study were offspring from the intercrosses

between the N1 generations. Genotyping was performed using DNA isolated from mice tails with primers specific for wild-type (WT) (P1 and P2) and for *Trib2* knockout (de-neo) alleles (P1 and P3), which gave products of 212 bp and 338 bp, respectively.

Supplementary tables

Table S1. Genotyping of Progenies from *Trib2* Heterozygote Crosses

	Genotype			
Age	+/+	+/-	-/-	Total
E18.5	9 (23%)	18 (46%)	12 (31%)	39
P1	12 (23%)	28 (54%)	12 (23%)	52
P2	9 (26%)	22 (65%)	3 (9%)	34
P10	57 (30%)	113 (59%)	22 (11%)	192

The neo-excised/Cre^{tg}-negative *Trib2*$^{+/-}$ heterozygous mice (backcrossed to C57BL/6 for 5 generations, N5) were intercrossed and the E18.5 embryos and pups at the age of postnatal day1, 2, or 10 (P1, p2 or p10) were collected for genotyping.

Table S2. Antibody conjugates and dilutions

**Conjugate**	**Antigen**	**Clone**	**Concentration**	**Dilution**	**Supplier**
PE	B220	RA3-6B2	0.2 mg/ml	1/100	eBioscience
FITC	CD3e	145-2C11	0.5 mg/ml	1/100	eBioscience
APC-Cy7	CD4	GK1.5	0.2 mg/ml	1/100	BioLegend

PE	CD8a	53-6.7	0.2 mg/ml	1/100	eBioscience
PE-Cy7	CD8a	53-6.7	0.2 mg/ml	1/100	BioLegend
APC	CD11b	M1/70	0.2 mg/ml	1/100	BioLegend
FITC	CD34	RAM34	0.5 mg/ml	1/100	eBioscience
Brilliant Violet 510	CD41	MWReg30	0.2 mg/ml	1/100	BioLegend
FITC	CD71	RI7217	0.5mg/ml	1/100	BioLegend
PE-Cy7	CD71	RI7217	0.2 mg/ml	1/100	BioLegend
Pacific Blue	CD105	MJ7/18	0.5 mg/ml	1/100	BioLegend
APC	CD150	TC15-12F12.2	0.2 mg/ml	1/100	BioLegend
APC-Cy7	c-kit (CD117)	2B8	0.2 mg/ml	1/100	BioLegend
PE-Cy7	FcgRII/III	93	0.2 mg/ml	1/200	BioLegend
FITC	Gr-1	RB6-8C5	0.5 mg/ml	1/100	eBioscience
APC	Sca-1	D7	0.5 mg/ml	1/100	BioLegend
PE-Cy5	Sca-1	D7	0.2 mg/ml	1/100	BioLegend
Alexa Fluor 700	Ter119	TER-119	0.5 mg/ml	1/100	BioLegend
PE	Ter-119	TER-119	0.2 mg/ml	1/100	BioLegend
PE	Rat IgG	Poly4054	0.2 mg/ml	1/200	BioLegend
APC-cy7	Streptavidin		0.2mg/ml	1/100	BioLegend

Table S3. Lineage cocktail composition (purified rat antibodies)

When used for Pronk's Myeloid-Erythroid progenitor staining protocol[1], anti-Ter119 antibody was excluded from this cocktail.

**Antigen**	**Clone**	**Concentration**	**Dilution**	**Supplier**

CD4	GK1.5	0.5 mg/ml	1/150	BD Biosciences
CD8a	53-6.7	0.5 mg/ml	1/150	BD Biosciences
B220	RA3-6B2	0.5 mg/ml	1/150	BD Biosciences
Gr-1	RB6-8C5	0.5 mg/ml	1/150	BD Biosciences
CD11b	M1/70	0.5 mg/ml	1/150	BD Biosciences
Ter119	Ter-119	0.5 mg/ml	1/150	BD Biosciences
IL-7 Ra	A7R34	0.5 mg/ml	1/150	BD Biosciences

Table S4. Antibodies used in myeloid progenitors staining (Akashi et. al. [2]**)**

**Conjugate**	**Antigen**	**Clone**	**Concentration**	**Dilution**	**Supplier**
PE	Rat IgG	Poly4054	0.2 mg/ml	1/200	BioLegend
APC	Sca-1	D7	0.5 mg/ml	1/100	BioLegend
APC-Cy7	c-kit (CD117)	2B8	0.2 mg/ml	1/100	BioLegend
PE-Cy7	FcgRII/III	93	0.2 mg/ml	1/200	BioLegend
FITC	CD34	RAM34	0.5 mg/ml	1/100	eBioscience

Table S5. Antibodies used in Myeloid-Erythroid progenitor staining (Pronk et. al. [1]**)**

**Conjugate**	**Antigen**	**Clone**	**Concentration**	**Dilution**	**Supplier**
PE	Rat IgG	Poly4054	0.2 mg/ml	1/200	BioLegend
APC-Cy7	c-kit (CD117)	2B8	0.2 mg/ml	1/100	BioLegend
PE-Cy7	FcgRII/III	93	0.2 mg/ml	1/100	BioLegend
PE-Cy5	Sca-1	D7	0.2 mg/ml	1/100	BioLegend
APC	CD150	TC15-12F12.2	0.2 mg/ml	1/100	BioLegend

Pacific Blue	CD105	MJ7/18	0.5 mg/ml	1/100	BioLegend
Brilliant Violet 510	CD41	MWReg30	0.2 mg/ml	1/100	BioLegend
Alexa Fluor 700	Ter119	TER-119	0.5 mg/ml	1/100	BioLegend
FITC	CD71	RI7217	0.5mg/ml	1/100	BioLegend

Table S6. Antibodies used in peripheral blood staining

**Conjugate**	**Antigen**	**Clone**	**Concentration**	**Dilution**	**Supplier**
PE	B220	RA3-6B2	0.2 mg/ml	1/100	eBioscience
FITC	CD3e	145-2C11	0.5 mg/ml	1/100	eBioscience
APC-Cy7	CD4	GK1.5	0.2 mg/ml	1/100	BioLegend
PE-Cy7	CD8a	53-6.7	0.2 mg/ml	1/100	BioLegend

Table S7. Primers used for semi-quantitative PCR (mouse)

**Gene**	**Primer**
Trib2	sense 5'-TAGCATGGGAACAAGACGTG-3'<br>antisense 5'-GCAATGCCAAGGTAGGTGTT-3'
SCL	sense 5'-CTGTTTGTGCAGGAGAGCAA-3'<br>antisense 5'-CAACAACTGGTCAGGCAGAA-3'
GATA2	sense 5'-TGCAACACACCACCCGATACC-3'<br>antisense 5'-CAATTTGCACAACAGGTGCCC-3'
GATA1	sense 5'-ATCAGCACTGGCCTACTACAGAG-3'<br>antisense 5'-GAGAGAAGAAAGGACTGGGAAAG-3'
C/EBPα	sense 5'-TAGGTTTCTGGGCTTTGTGG-3'

	antisense 5’-AGCATAGACGTGCACACTGC-3’
EpoR	sense 5’-GGACACCTACTTGGTATTGG -3’<br><br>antisense 5’-GACGTTGTAGGCTGGAGTCC-3’
β-actin	sense 5’-AGAGGGAAATCGTGCGTGAC-3’<br><br>antisense 5’- CAATAGTGATGACCTGGCCGT-3’

Table S8. Primers used in the QuikChange® mutagenesis of murine Trib2

Mutated nucleotides are highlighted in gray. Reverse primers were exactly complementary to the forward primers.

Mutant	Forward primer
mTrib2-K177R	5’-GTGCTGCGTGACCTC**CGT**CTGCGGAAATTTATC-3’
mTrib2-KDM	5’-CTGCGTGACCTCAAGC**CCGA**GAA**C**TTTATCTTCAAGG-3’
mTrib2-VPM	5’-GCTAAAGAGGCGTGTG**CT**CAGCTGG**CTG**C**T**GACGTCAACATGG-3’

Table S9. Primers used for mouse genotyping

**Primer name**	**Primer**
P1	5’-GGCTAGTTCTTAACTCTTCCTCCA-3’
P2	5’-TCCCATATCTCGCTATTGTGATAG-3’
P3	5’-CATTCGCCATTCAGGCTGCGCAAC-3’
P4	5’-AAGTCCTCTAGGTTCTCAGTGTTC- 3’
P5	5’-CATTGCATCAGCCATGATGGATAC-3’
P6	5’-CCATTACCAGTTGGTCTGGTGTCA-3’
Cre-forward primer	5’-TGCCACGACCAAGTGACAGC-3’

Cre-reverse primer	5'-CCTTAGCGCCGTAAATCAATCG-3'

Supplementary Figures

Figure S1. The strategy for targeted disruption of the *Trib2* gene in the mouse genome.

(A) The schematic representation of the architectures for the wild-type (wt) *Trib2* genomic allele, targeting vector, floxed-neo targeted allele and the de-neo targeted (mt) allele are shown together with some relevant restriction sites (E, *Ec*oRV; K, *Kpn*I). The numbered boxes represent exons of the *Trib2* gene. The LacZ and Neo gene cassettes are shown with broad arrows. The locations of genotyping primers, including P1, P2, P3, P4, P5 and P6, are all indicated. The 5' (black bar) and 3' (red bar) probes and the predicted lengths of *Ec*oRV and *Kpn*I restriction fragments in Southern blot analyses for the wt *Trib2* allele and the targeted *Trib2* allele are shown as indicated. (B, C) Southern blot analysis of the G418-resistant recombinant ES cell clone harboring the "floxed-neo targeted allele". Genomic DNA extracted from ES cell clones (Lane 1, parental; Lane2: clone #177) was digested with *Eco*RV and probed with the 5'probe (B) or digested with *Kpn*I and probed with the 3' probe (C). The predicted signals for the wild-type (wt) and floxed-neo targeted allele (f-neo) are as indicated. (D) Representative genotypic analysis of 3-week old mice harboring the WT (+) or KO allele (-) of

the *Trib2* gene from a *Trib2*$^{+/-}$ intercross. Genotyping was performed by PCR using primers P1 and P2 for the wild-type (WT, 212 bp), and P1 and P3 for the targeted allele (de-neo, 338 bp). (E) Representative genotypic analysis of 3-week old *Trib2*$^{+/-}$ mice harboring the targeted locus with floxed-neo or without neo (de-neo). To generate *neo*-resistance cassette-free *Trib2* knockout mice, *Trib2*$^{+/-}$ female mice carring the *neo*^{r} gene were crossed to *EIIa Cre* transgenic (*Cre*^{tg}) male mice to remove the *neo*^{r} gene from the targeted locus. Genotyping was performed by PCR using primers P5 and P4 for the floxed-neo locus (1kb), and P6 and P4 for the de-neo locus (0.6kb). (F) Detection of endogenous Trib2 protein expression in wt (+/+), but not in knockout (-/-) mice. Activated splenic CD4^{+} T cells with the indicated genotypes were deprived of IL-2 for 12h before total protein lysates were analyzed by Western blotting using Trib2 or β-actin specific antibodies. NS: non-specific signal.

Figure S2. Similar total splenocyte and erythroblast numbers between WT and KO mice with or without PHZ treatment.

Hemolytic anemia was induced by two consecutive injections of PHZ (PBS in the control group) at days 1 and 2 (50 mg/kg mice). Five days after the first PHZ injection, mice were sacrificed, and cellularity as well as erythroblast differentiation were assessed in the spleen. (A) Photographs of spleens from PBS or PHZ-treated *Trib2*$^{+/+}$ and *Trib2*$^{-/-}$ mice. Representative CD71/TER119 FACS profiles (B) and statistical analysis (C) of data derived from (B) are

shown. The presented values are means ± SEM from 6–10 mice per group. Total cell number (D) and total Ter119^{+} cell number (E) of spleens from *Trib2*$^{+/+}$ and *Trib2*$^{-/-}$ mice in control or PHZ treatment groups are also shown.

Figure S3. Purification of LKS^{+}, CMPs, GMPs, MEPs and erythroblasts by flow cytometry from total bone marrow cells.

(A) Step-wise purification of various hematopoietic early progenitor compartments (shown on the right) from total bone marrow cells by sorting the cells stained with specific antibodies against C-kit, Sca1, FcγR, CD34 and lineage (Lin) markers, as described by Weissman[2]. (B) Isolation of various erythroblastic compartments (shown on the right) by sorting the cells stained with erythroid surface markers CD71 and Ter119, as defined by Socolovsky et al.[3]. LKS+, Lin^{-}c-kit^{+}ScaI^{+} cell; CMP, common myeloid progenitor; GMP, granulocyte/macrophage progenitor; MEP, megakaryocyte/erythrocyte progenitor; R1, proerythroblast; R2, basophilic erythroblast; R3, polychromatic erythroblast; R4, orthochromatic erythroblast. R1 is the most primitive and R4 is the most differentiated.

Figure S4. *Trib2* deletion reduces the erythrocytes in bone marrow. (A) Reduced erythroblasts in *Trib2*$^{-/-}$ bone marrow. Representative CD71/TER119 FACS profiles of

bone marrow from *Trib2*$^{+/+}$ and *Trib2*$^{-/-}$ mice (backcrossed to B6, N5). (B) Statistical data analysis of (A). Values were derived from CD71/TER119 FACS profiles and are presented as the mean ± SEM for 6 mice per genotype. (C, D) Reduced MEP population in *Trib2*$^{-/-}$ bone marrow (backcrossed to B6, N5). Total bone marrow cells were first subjected to gating of lineage markers (Lin) expression. The Lin^{-} fraction was then analyzed for Sca1 and c-kit expression (C, left panels). Lin^{-}c-Kit^{+}ScaI^{-} (LKS^{-}) cells were further analyzed for CD34 and FcγRII/III expression (C, right panels). The percentage of each population is shown. Quantification of each CMP, GMP and MEP population, together with statistical data, are shown in (D). The graphs show the mean and S.E.M., *n*=6 for each group.

Figure S5. Representative photographs of mixed CFU, BFU-E, CFU-GM, CFU-M and CFU-G colonies. Isolated CMPs were cultured in MethoCult M3434 methylcellulose-based medium for 10 days and phase contrast (left panel) images were taken before and May-Grünwald-Giemsa staining (right panel) images were taken after cytospin preparation. The scale bar is 50 μm.

References for supplementary information:

1 Pronk, C. J. *et al.* Elucidation of the phenotypic, functional, and molecular topography of a myeloerythroid progenitor cell hierarchy. *Cell Stem Cell* **1**, 428-442 (2007).

2 Akashi, K., Traver, D., Miyamoto, T. & Weissman, I. L. A clonogenic common myeloid progenitor that gives rise to all myeloid lineages. *Nature* **404**, 193-197 (2000).

3 Socolovsky, M. *et al.* Ineffective erythropoiesis in Stat5a(-/-)5b(-/-) mice due to decreased survival of early erythroblasts. *Blood* **98**, 3261-3273 (2001).

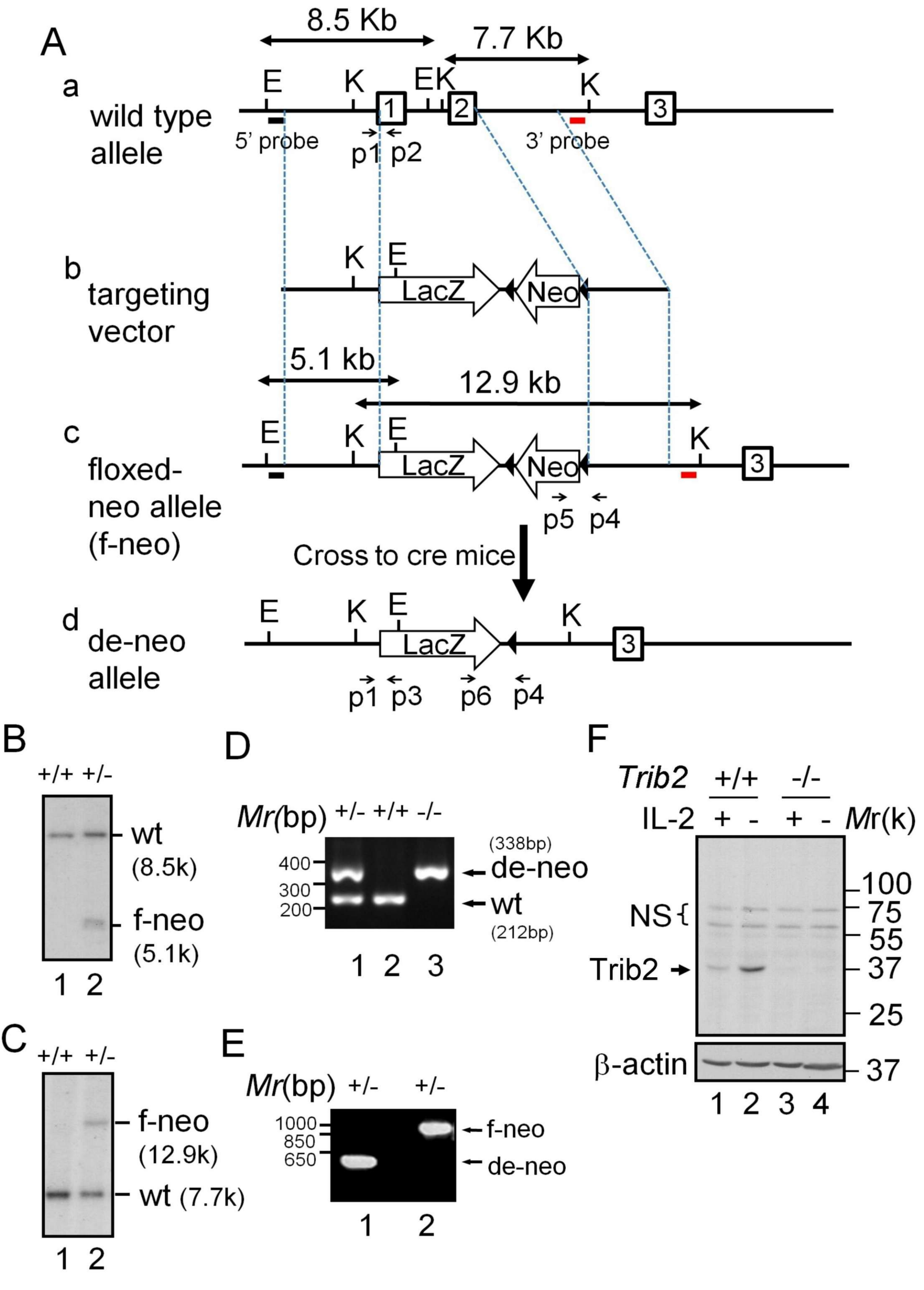
A
8.5 Kb
7.7 Kb
a
wild type allele
E
K
1
EK
2
K
3
5' probe
p1 p2
3' probe
b
targeting vector
K
E
LacZ
Neo
5.1 kb
12.9 kb
c
floxed-neo allele (f-neo)
E
K
E
LacZ
Neo
K
3
p5 p4
Cross to cre mice
d
de-neo allele
E
K
E
LacZ
K
3
p1 p3 p6 p4
B
+/+ +/-
wt (8.5k)
f-neo (5.1k)
1 2
C
+/+ +/-
f-neo (12.9k)
wt (7.7k)
1 2
D
Mr(bp) +/- +/+ -/-
400
300
200
(338bp)
de-neo
wt
(212bp)
1 2 3
E
Mr(bp) +/- +/-
1000
850
650
f-neo
de-neo
1 2
F
Trib2 +/+ -/-
IL-2 + - + -
Mr(k)
100
75
55
37
25
NS
Trib2
β-actin
37
1 2 3 4

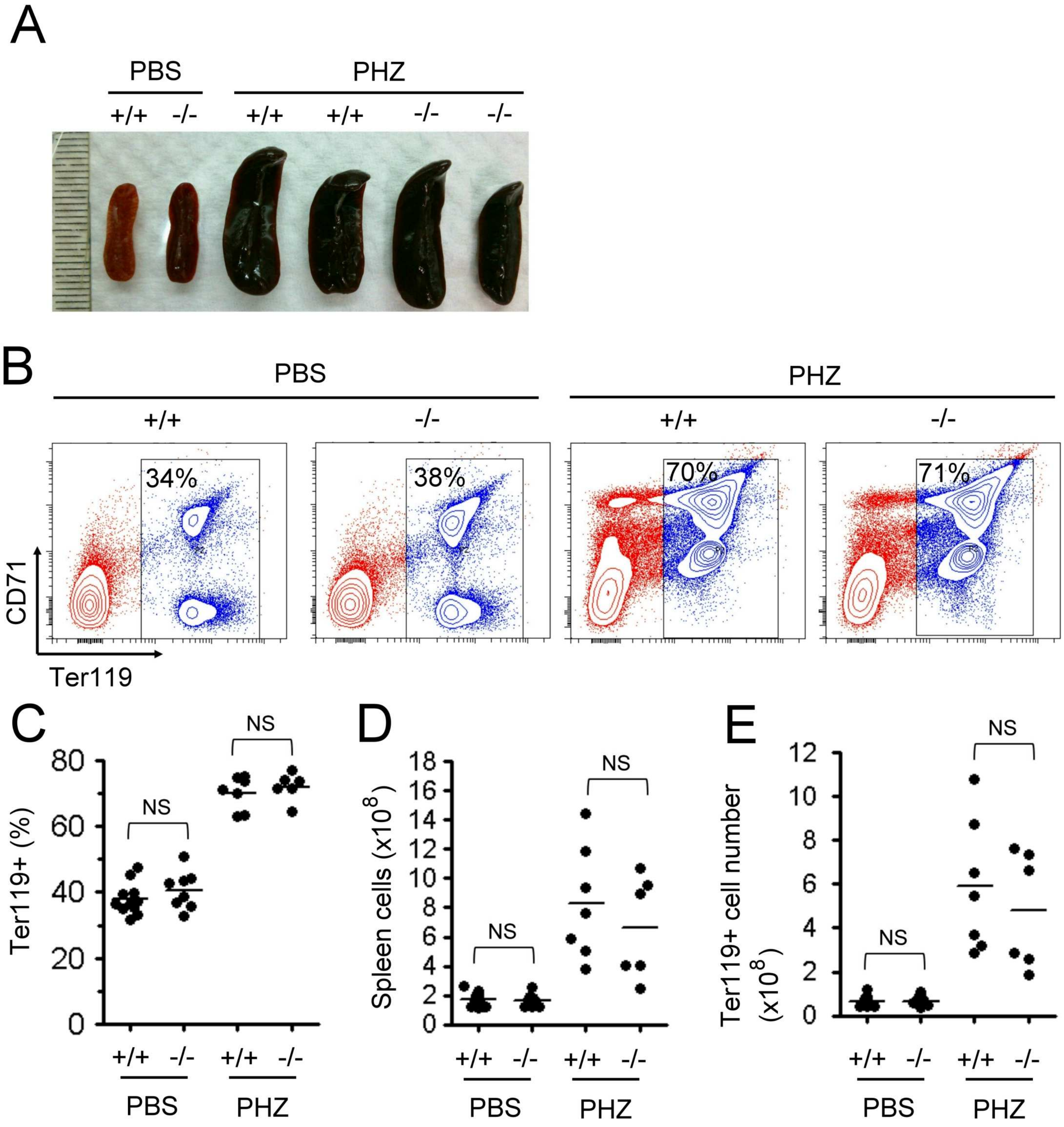
A
PBS
PHZ
+/+
-/-
+/+
+/+
-/-
-/-
B
PBS
PHZ
+/+
-/-
+/+
-/-
34%
38%
70%
71%
CD71
Ter119
C
Ter119+ (%)
80
60
40
20
0
NS
NS
+/+
-/-
+/+
-/-
PBS
PHZ
D
Spleen cells (x10^8)
18
16
14
12
10
8
6
4
2
0
NS
NS
+/+
-/-
+/+
-/-
PBS
PHZ
E
Ter119+ cell number (x10^8)
12
10
8
6
4
2
0
NS
NS
+/+
-/-
+/+
-/-
PBS
PHZ

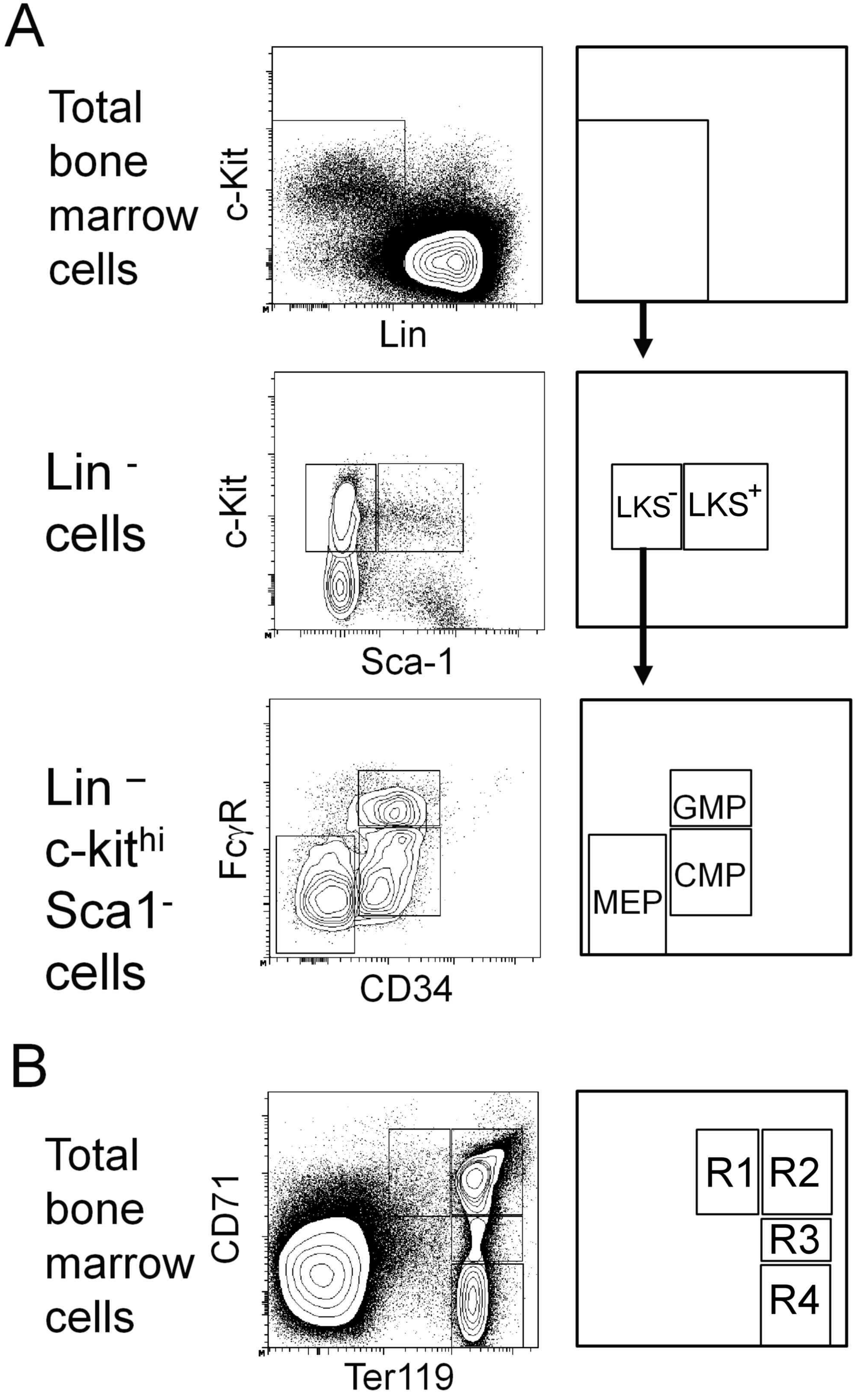
A
Total bone marrow cells
c-Kit
Lin
Lin - cells
c-Kit
Sca-1
LKS-
LKS+
Lin – c-kithi Sca1- cells
FcγR
CD34
GMP
CMP
MEP
B
Total bone marrow cells
CD71
Ter119
R1
R2
R3
R4

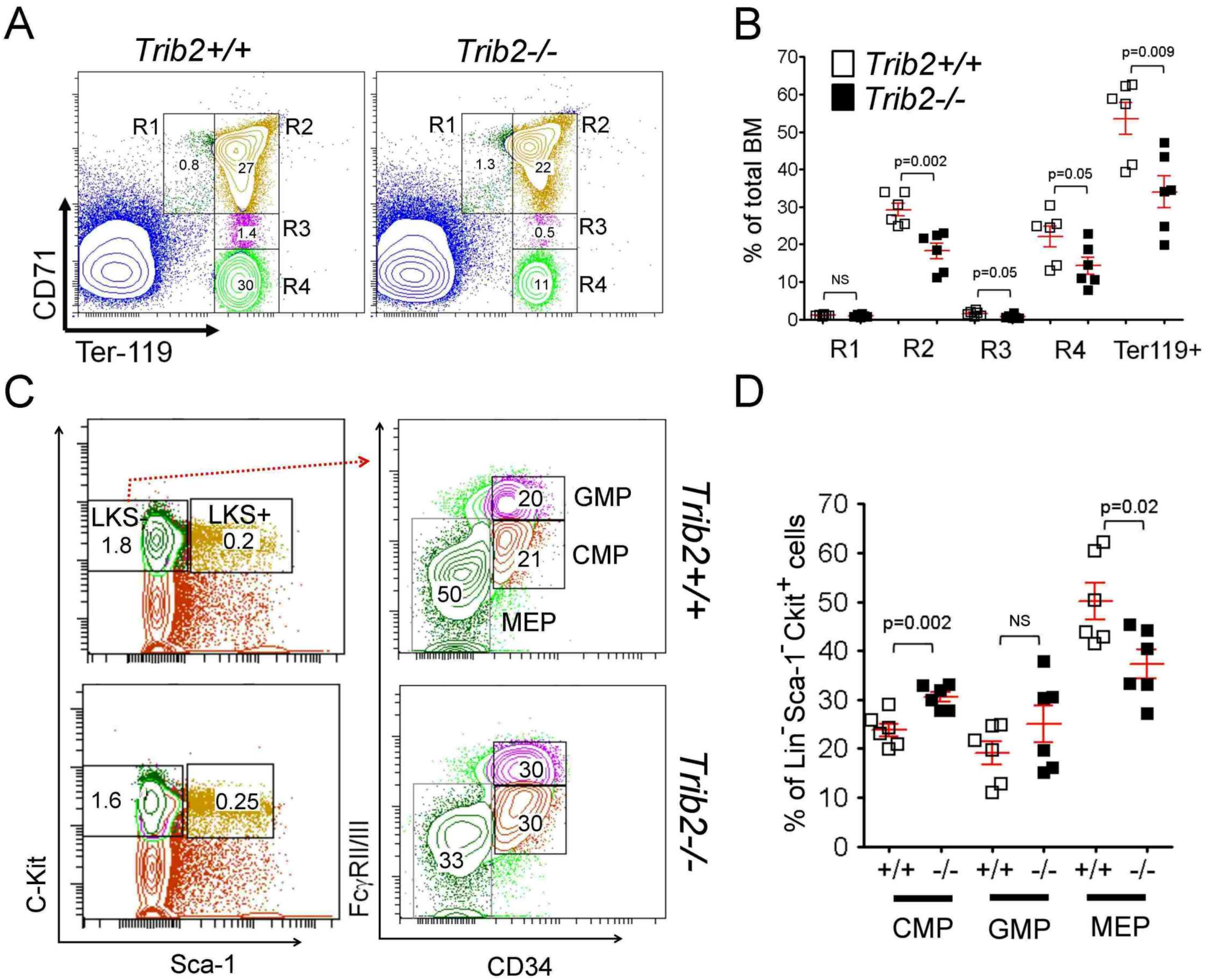

A
Trib2+/+
Trib2-/-
R1
R2
R3
R4
0.8
27
1.4
30
1.3
22
0.5
11
CD71
Ter-119
B
Trib2+/+
Trib2-/-
% of total BM
NS
p=0.002
p=0.05
p=0.05
p=0.009
R1
R2
R3
R4
Ter119+
C
LKS-
1.8
LKS+
0.2
1.6
0.25
20
GMP
21
CMP
50
MEP
30
30
33
C-Kit
Sca-1
FcγRII/III
CD34
D
% of Lin⁻Sca-1⁻Ckit⁺ cells
p=0.002
NS
p=0.02
+/+
-/-
CMP
GMP
MEP

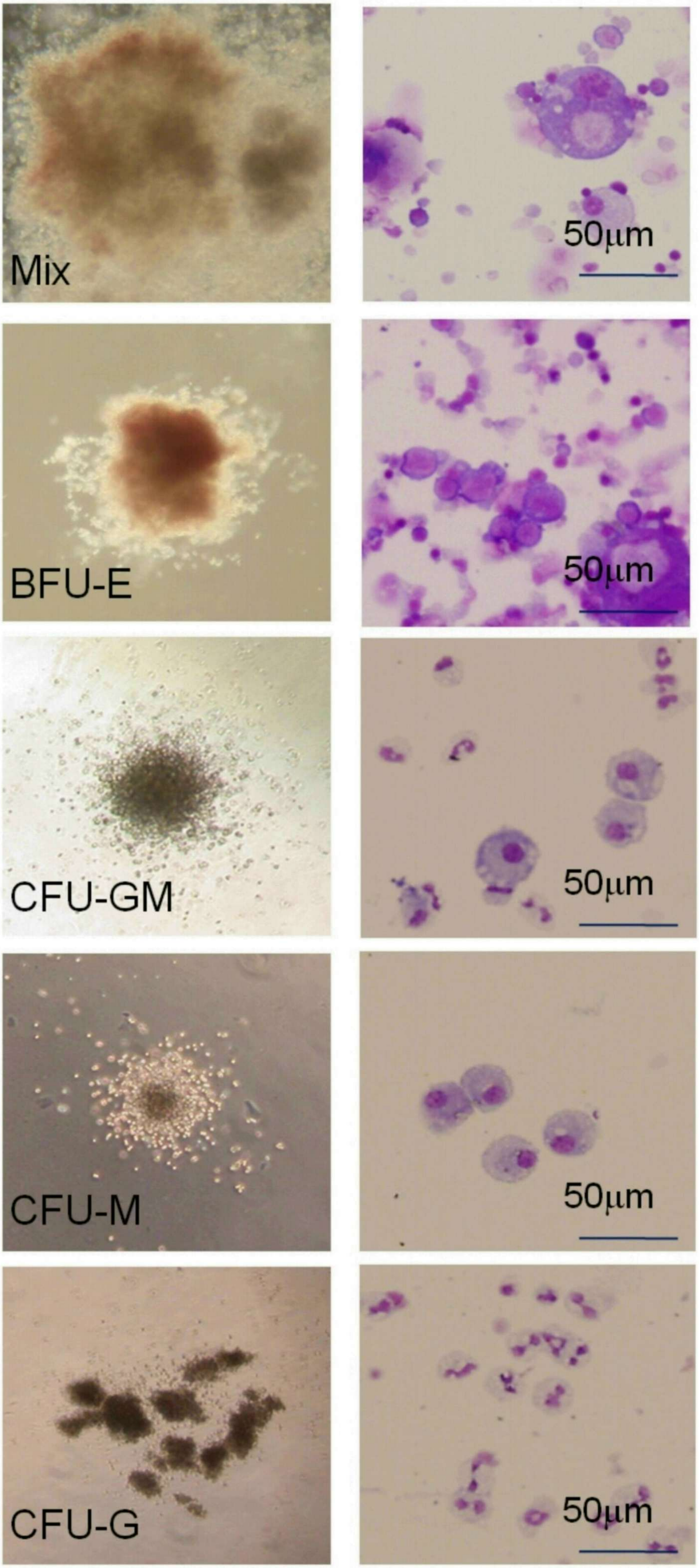
Mix
50μm
BFU-E
50μm
CFU-GM
50μm
CFU-M
50μm
CFU-G
50μm
